# Design and Synthesis of Bisulfone-Linked Two-Dimensional Conjugated Microporous Polymers for CO_2_ Adsorption and Energy Storage

**DOI:** 10.3390/molecules28073234

**Published:** 2023-04-04

**Authors:** Mohamed Gamal Mohamed, Siang-Yi Chang, Moshin Ejaz, Maha Mohamed Samy, Aya Osama Mousa, Shiao-Wei Kuo

**Affiliations:** 1 Department of Materials and Optoelectronic Science, College of Semiconductor and Advanced Technology Research, Center of Crystal Research, National Sun Yat-Sen University, Kaohsiung 804, Taiwan; shiang120321@gmail.com (S.-Y.C.);; 2 Chemistry Department, Faculty of Science, Assiut University, Assiut 71515, Egypt; 3 Department of Medicinal and Applied Chemistry, Kaohsiung Medical University, Kaohsiung 807, Taiwan

**Keywords:** dibenzo[g,p]chrysene, conjugated microporous polymers, Sonogashira–Hagihara coupling reaction, CO_2_ capture, supercapacitors

## Abstract

We have successfully synthesized two types of two-dimensional conjugated microporous polymers (CMPs), Py-BSU and TBN-BSU CMPs, by using the Sonogashira cross-coupling reaction of BSU-Br_2_ (2,8-Dibromothianthrene-5,5′,10,10′-Tetraoxide) with Py-T (1,3,6,8-Tetraethynylpyrene) and TBN-T (2,7,10,15-Tetraethynyldibenzo[g,p]chrysene), respectively. We characterized the chemical structure, morphology, physical properties, and potential applications of these materials using various analytical instruments. Both Py-BSU and TBN-BSU CMPs showed high thermal stability with thermal decomposition temperatures (T_d10_) up to 371 °C and char yields close to 48 wt%, as determined by thermogravimetric analysis (TGA). TBN-BSU CMPs exhibited a higher specific surface area and porosity of 391 m^2^ g^−1^ and 0.30 cm^3^ g^−1^, respectively, due to their large micropore and mesopore structure. These CMPs with extended π-conjugated frameworks and high surface areas are promising organic electroactive materials that can be used as electrode materials for supercapacitors (SCs) and gas adsorption. Our experimental results demonstrated that the TBN-BSU CMP electrode had better electrochemical characteristics with a longer discharge time course and a specific capacitance of 70 F g^−1^. Additionally, the electrode exhibited an excellent capacitance retention rate of 99.9% in the 2000-cycle stability test. The CO_2_ uptake capacity of TBN-BSU CMP and Py-BSU CMP were 1.60 and 1.45 mmol g^−1^, respectively, at 298 K and 1 bar. These results indicate that the BSU-based CMPs synthesized in this study have potential applications in electrical testing and CO_2_ capture.

## 1. Introduction

While digital life brings comfort, it also presents difficulties that people must consider. This energy source and storage support the advent of electronic products, and the energy relies primarily on the supply of the natural environment [1,2,3,4,5,6,7]. Renewable energy sources include solar and wind power, ocean energy, hydropower, and biomass; limited energy sources come from industrial fossil fuels such as coal, natural gas, and oil [8,9,10,11,12,13,14,15]. The pursuit of green environmental protection without affecting the life experience of most consumers, or how to ensure the supply and demand of energy while improving economic effects, is a heavy part of technology today. An increasingly well-known energy storage device to deal with this situation is the supercapacitor (SC), which has been studied since the 19th century [16,17,18,19,20,21,22,23,24,25]. SCs were developed for the same purpose as secondary batteries and conventional capacitors. They are all used for the convenience of energy storage, but supercapacitors have fewer limitations and wider prospects than the other two [26,27,28,29,30]. For example, its excellent reaction kinetics, higher power density, and gradually improved energy density make it more suitable for charging and discharging [26,27,28,29,30]. In addition, the improved cycle life and wider operating range of SCs allow them to operate stably and continuously [31,32,33,34,35]. Currently, it has achieved practical penetration in many fields, including mobile wearable devices, smart electronic appliances, electric transportation vehicles, medical treatment, and the defense industry [36,37,38,39,40]. SCs can be preliminarily divided into electric double-layer capacitors (EDLCs), pseudocapacitors (PCs), and hybrid supercapacitors (HSCs) based on their charge storage mechanism [36,37,38,39,40]. The energy storage principle of EDLCs focuses on electrostatic adsorption between the electrodes and electrolytes. EDLCs are highly efficient and reversible owing to their charge-physical adsorption behavior. Nevertheless, decisive factors concerning the reactivity of electrochemical capacitors are the properties of the electrolyte and electrodes [41,42,43,44,45,46,47,48,49,50]. The electrode materials used in EDLCs are primarily metal oxides, carbon-based materials, or conducting polymers [51,52,53,54,55,56,57,58,59]. We have investigated many modifications based on the molecular design or synergistic effects of conducting polymers to enhance their competitiveness in the market.

Among these, covalently linked and highly cross-linked conjugated microporous polymers (CMPs) are suitable for this concept [60,61,62,63,64]. The structural diversity of π systems and chemically reactive groups coupled with chemical stability and electrical conductivity are attractive advantages of CMPs. Specifically, a framework rich in micropores contributes to excellent surface conditions [60,61,62,63,64]. On this premise, the introduction of thianthrene-5,5′,10,10′-tetraoxide as a matrix in CMP enhances charge injection/transport performance and inter-bond interactions [65,66]. This group is beneficial for making the sample highly rigid [65,66]. In contrast, the participation of aromatic systems in Py-T and TBN-T units improves the conjugation properties of the materials. The pyrene contained in the former structure was connected by four benzene rings, which facilitated π-π stacking. The distribution of electrons exhibited by this state has led to great progress in the conduction performance of organic electrochemical substances. Under these circumstances, the continuous conjugated structure of the pyrene molecule can also reduce steric hindrance and change the flexibility of the chain [67,68,69,70,71]. For the same reason, TBN-T was chosen as one of the backbones in another CMP synthesis reaction. The TBN structure of the monomer center is upgraded to be connected by six benzene rings, which can improve the electrical properties of the material without sacrificing the rigidity of the material [72,73,74]. This has a positive effect on SCs.

**Scheme 1:**
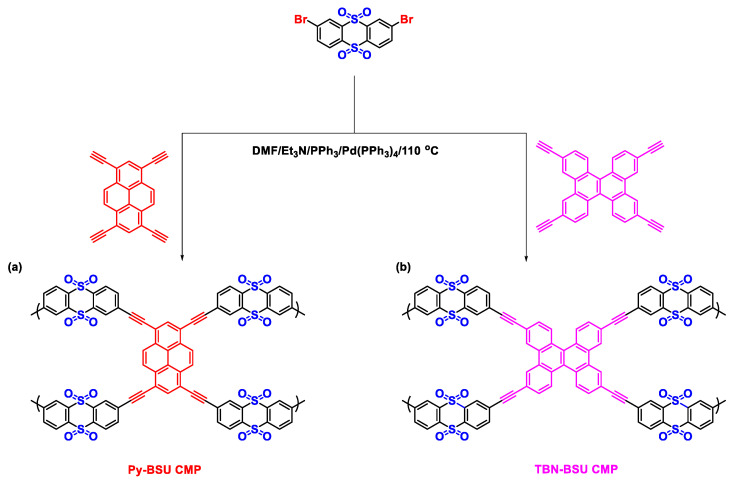
Synthetic route for (**a**) Py-BSU CMP and (**b**) TBN-BSU CMP.

In summary, we believe that BSU CMPs, with practical connotations, are highly feasible to become a potential stock for emerging developments. We believe that this is the first investigation into CMP materials with a bisulfone (BSU) moiety for applications involving gas uptake and energy storage. Herein, a facile and efficient Sonogashira cross-coupling reaction was used to construct and prepare two 2D-CMP-containing bisulfone moieties in high yields (Py-BSU and TBN-BSU CMPs) by the reaction of brominated thianthrene-5,5′,10,10′-tetraoxide with different ethynylpyrene (Py-T) and ethynyldibenzo[g,p]chrysene (TBN-T) molecules in DMF/Et_3_N as solvents and Pd catalysts (Scheme 1). Solid-state NMR (ssNMR) and FTIR investigations supported the formation of Py-BSU and TBN-BSU CMPs, and PXRD, BET, SEM, EDS-SEM mapping, TEM, and TGA also investigated their morphology and porosity. Due to their good properties, such as high thermal stability, extended π-conjugated frameworks, and good BET surface areas, these materials are applied in CO_2_ capture and supercapacitors applications. The electrochemical experiments show that the Py-BSU CMP and TBN-BSU CMP electrodes in the three-electrode system exhibited specific capacitances of 38 and 70 F g^−1^, respectively, and an excellent capacitance retention rate of up to 99% in the 2000-cycle stability test. Furthermore, the TBN-BSU CMP sample exhibited high CO_2_ uptake (1.60 mmol g^−1^) due to the high surface area, pore volume, and tunable pore size of TBN-BSU CMP. According to these results, the BSU-based CMP prepared in this study has novel and accessible application potential in gas adsorption and electrical testing.

## 2. Results and Discussion

### 2.1. Synthesis and Characterization of Synthesized Monomers, Py-BSU and TBN-BSU CMPs

The BSU-Br_2_ monomer was prepared in two steps. The THT molecule was brominated in a neat Br_2_ solution in AcOH to afford THT-Br_2_ as a white solid (Scheme S1). THT-Br_2_ was then reacted with H_2_O_2_ solution in AcOH solution at 90 °C to produce BSU-Br_2_ as a white solid (Scheme S2). The FTIR pattern of BSU-Br_2_ (Figure S4) showed peaks at 3064 cm^−1^ for the aromatic rings and 1171 cm^−1^ for the SO_2_ group, indicating the successful formation of BSU-Br_2_ from THT-Br_2_. Furthermore, the ^1^H and ^13^C NMR data confirmed the chemical structure of BSU-Br_2_ (Figures S5 and S6). The FTIR and NMR results for other synthesized monomers in this study, such as THT-Br_2_, Py-Br_4_, Py-TMS, Py-T, TPE, TPE-Br_4_, TBN-Br_4_, and TBN-TMS, have been provided and discussed in detail in the experimental section (Figures S1–S3 and S7–S25). The signals at 3279, 3065, 2186, and 1618 cm^−1^ are attributed to the absorption characteristics for ≡C–H, aromatic C–H, C≡C, and C=C stretching in the Py-T structure (Figure S11). The ^1^H NMR pattern (Figure S12) of Py-T peaked at 8.68, 8.38, and 3.67 ppm, corresponding to the phenyl and alkynyl groups, respectively. In addition, the aromatic carbon resonances appeared in the range 133.80–127.80, 84.50, and 82.00 ppm in the ^13^C NMR spectrum (Figure S13) of Py-T due to the presence of aromatic and alkynyl units. As shown in Schemes S5–S7, there were three steps during the preparation of the TBN-T monomer. In dry nitromethane and DCM, TPE-Br_4_ was reacted with anhydrous FeCl_3_ to produce a yellow powder of TBN-Br_4_. To prepare TBN-TMSA, a TBN-Br_4_ precursor was combined with (trimethylsilyl)acetylene (TMSA) in the presence of CuI with diethylamine as the solvent. The treatment of TBN-TMSA with K_2_CO_3_ in methanol and DCM to produce a yellow solid allowed for the effective synthesis of TBN-T. The peaks in the FTIR spectrum of TBN-T (Figure S26) at 3291, 3057, 2106, and 1604 cm^−1^ were attributed to C≡C-H, aromatic C-H, C≡C, and C=C stretching, respectively. The proton signal was observed in the ^1^H NMR spectra of TBN-T (Figure S27) at 3.30 ppm for C≡C-H. In addition, the presence of aromatic rings and C≡C units in the TBN-T compound caused carbon peaks at 135.70–101.60, 84.21, and 78.87 ppm, as seen in the ^13^C-NMR spectrum (Figure S28).

**Figure 1:**
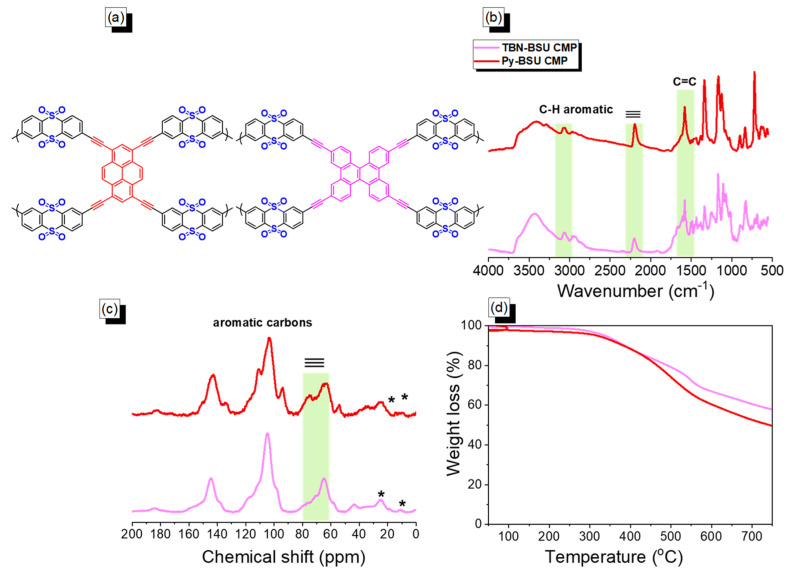
(**a**) Schematic diagram. (**b**) FTIR spectra, (**c**) SSNMR, and (**d**) TGA curves of Py-BSU CMP and TBN-BSU CMP. * the side band of solid-state nuclear magnetic resonance spectroscopy (NMR).

As shown in Scheme 1, Py-BSU CMP (Scheme 1a) and TBN-BSU CMP (Scheme 1b) were prepared by reacting BSU-Br_2_ with Py-T and TBN-T in a mixture of DMF/Et_3_N for 3 days to afford dark red powder for Py-BSU CMP and yellow solid for TBN-BSU CMP. The solubility test in different organic solvents (NMP, DMSO, DMF, MeOH, EtOH, DCM, and THF) revealed the insolubility of these materials compared to their corresponding monomers (BSU-Br_2_, Py-T, and TBN-T) and confirmed that both BSU-CMP frameworks had a high cross-linking density. The chemical characteristics of Py-BSU and TBN-BSU CMPs were identified by Fourier transform infrared spectroscopy and solid-state ^13^C nuclear magnetic resonance spectroscopy (ssNMR). A schematic of the corresponding structure is shown in Figure 1a. The absorption bands in Py-BSU CMP and TBN-BSU CMP around 3070 and 1580 cm^−1^ in the FTIR profiles (Figure 1b) measured at room temperature revealed the stretching of C-H and C=C in aromatic units, and the stretching absorption band of the C≡C bond appeared at 2200 cm^−1^. The appearance of absorption bands centered at 1167 cm^−1^ for Py-BSU CMP and 1177 cm^−1^ for TBN-BSU CMP corresponds to the SO_2_ unit in their framework (Figure 1b). The broad peaks at 95–115 ppm in the ssNMR spectra (Figure 1c) represent the signals of aromatic carbon atoms in the Py-BSU CMP and TBN-BSU CMP structures. Internal -C≡C- units in both Py-BSU CMP and TBN-BSU CMP were centered at 74.80 and 64.04 ppm, respectively [75,76,77]. The above results confirmed that Py-BSU CMP and TBN-BSU CMP were successfully constructed through a coupling reaction. In addition, the thermogravimetric analysis (TGA) in Figure 1d and Table 1 demonstrates the good thermal stability of the BSU-CMP materials under a nitrogen atmosphere, which is due to the high degree of cross-linking of the BSU-CMP materials after the Sonogashira cross-coupling reaction. The 5% weight loss (T_d5_) of Py-BSU CMP and TBN-BSU CMP occurred at 320 and 338 °C, 10% weight loss (T_d10_) of Py-BSU CMP and TBN-BSU CMP occurred at 383 and 386 °C, respectively, and the char yields at 800 °C were 48 and 56 wt%, respectively.

**Figure 2:**
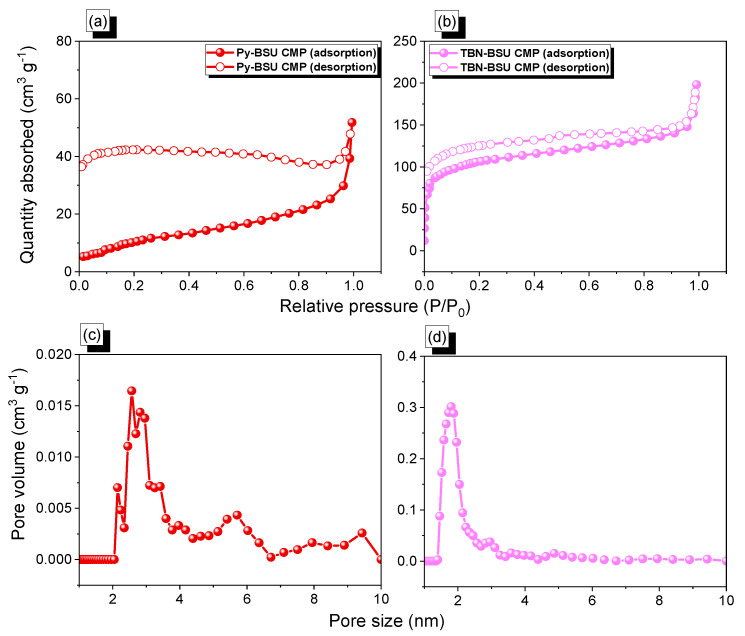
(**a**,**b**) N_2_ adsorption/desorption isotherms and (**c**,**d**) pore size distribution profiles of (**a**,**c**) Py-BSU CMP and (**b**,**d**) TBN-BSU CMP.

The porosity behavior of Py-BSU CMP and TBN-BSU CMP was determined using the Brunauer-Emmett-Teller (BET) theory, and the specific surface area and pore characteristics were confirmed through nitrogen adsorption/desorption analysis at 77 K. Both Py-BSU CMP and TBN-BSU CMP were vacuum degassed at 150 °C for 8 h to eliminate the huge effect caused by water and other gases in the gas adsorption experiment. According to the IUPAC classification, the Py-BSU CMP in Figure 2a exhibits a type V isotherm with H_2_ hysteresis loops that reflect typical ink bottle holes with uneven pore size distributions or close-packed interstitial pores of spherical particles. While TBN-BSU CMP in Figure 2b exhibits a type I isotherm with an H_1_ hysteresis loop, the surge in the region of low relative pressure (<0.02) confirms that this material has a large number of micropores. The specific surface areas of Py-BSU CMP and TBN-BSU CMP were 42 and 391 m^2^ g^−1^. Their corresponding total pore volumes are 0.07 and 0.30 cm^3^ g^−1^, respectively. In addition, we estimated the pore size distribution (PSD) of Py-BSU CMP and TBN-BSU CMP from the N_2_ adsorption isotherm using the nonlocal density functional theory (NLDFT). The results show that the average pore diameters of Py-BSU CMP (Figure 2c) and TBN-BSU CMP (Figure 2d) were approximately 2.57 and 1.80 nm.

**Figure 3:**
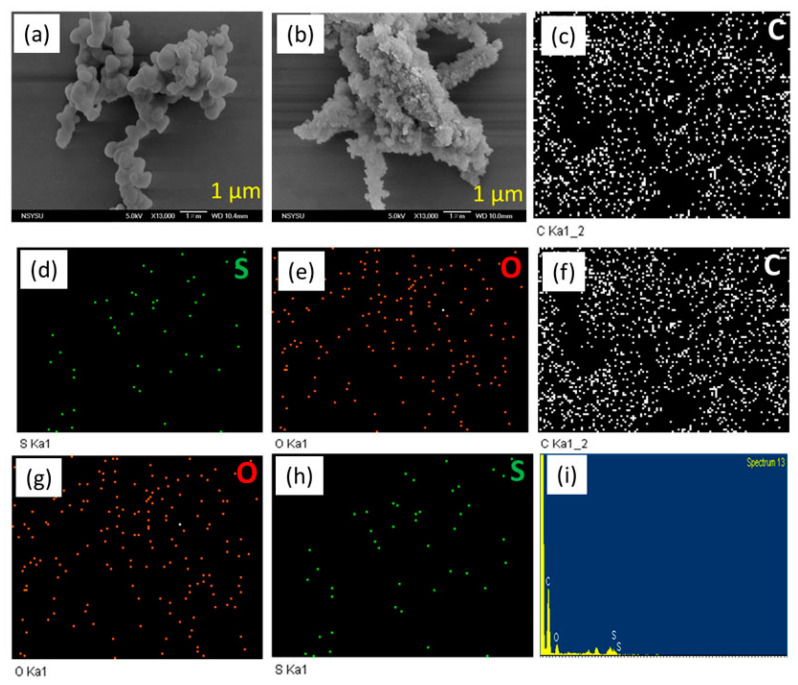
SEM images (**a**,**b**) and EDS-SEM mapping (**c**–**i**) of Py-BSU CMP (**a**,**c**–**e**) and TBN-BSU CMP (**b**,**f**–**i**).

The spatial ordering of Py-BSU CMP and TBN-BSU CMP was determined by X-ray diffraction analysis in the angular (2θ) range of 5–50°. Crystalline peaks were not observed in the XRD results, and the broad peaks in Figure S29 show the amorphous features of Py-BSU CMP and TBN-BSU CMP. The powder morphologies by SEM images of Py-BSU CMP and TBN-BSU CMP are shown in Figure 3a,b, respectively. The former nanosheets agglomerated slightly; the latter cluster phenomenon occurs because of the extremely small size of spherical nanoparticles. Furthermore, the presence of carbon (C), oxygen (O), and sulfur (S) atoms in both Py-BSU CMP and TBN-BSU CMP were confirmed through EDS-SEM mapping (Figure 3c–i). The weight contents of C, O, and S in Py-BSU CMP were 78.80, 16.82, and 4.38%, respectively (Figure S30a). In TBN-BSU CMP, the weight contents were 70.9, 26.64, and 2.46% for C, O, and S atoms, respectively (Figure S30b). The TEM images (Figure S31) of both Py-BSU CMP and TBN-BSU CMP show that the structure was amorphous and that nanometer-scale holes were distributed uniformly.

**Figure 4:**
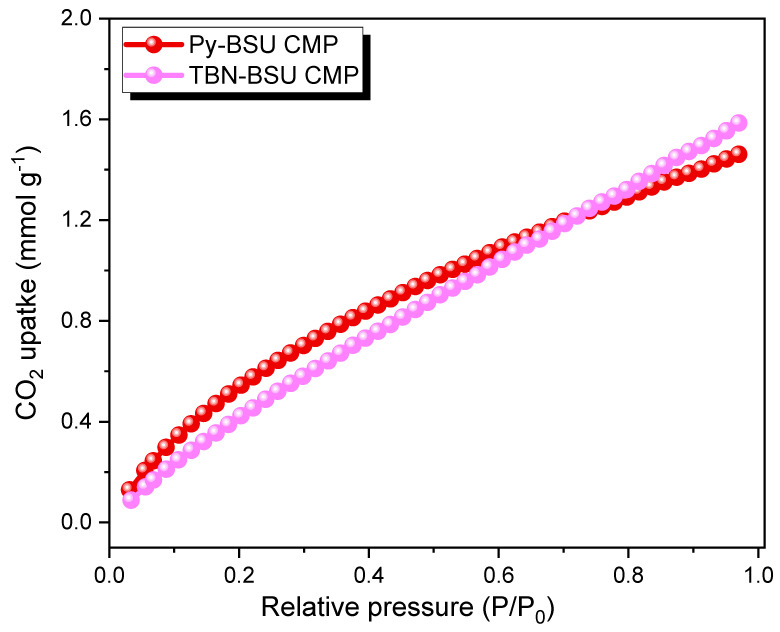
CO_2_ uptake of Py-BSU CMP and TBN-BSU CMP was recorded at 298 K.

The CO_2_ capacities of TBN-BSU CMP and Py-BSU CMP were 1.6 and 1.45 mmol g^−1^, respectively, as displayed in Figure 4. The excellent performance of TBN-BSU CMP for CO_2_ uptake is due to its high pore volume, BET surface area, and tunable pore size.

### 2.2. Electrochemical Measurements of Py-BSU CMP and TBN-BSU CMP

**Figure 5:**
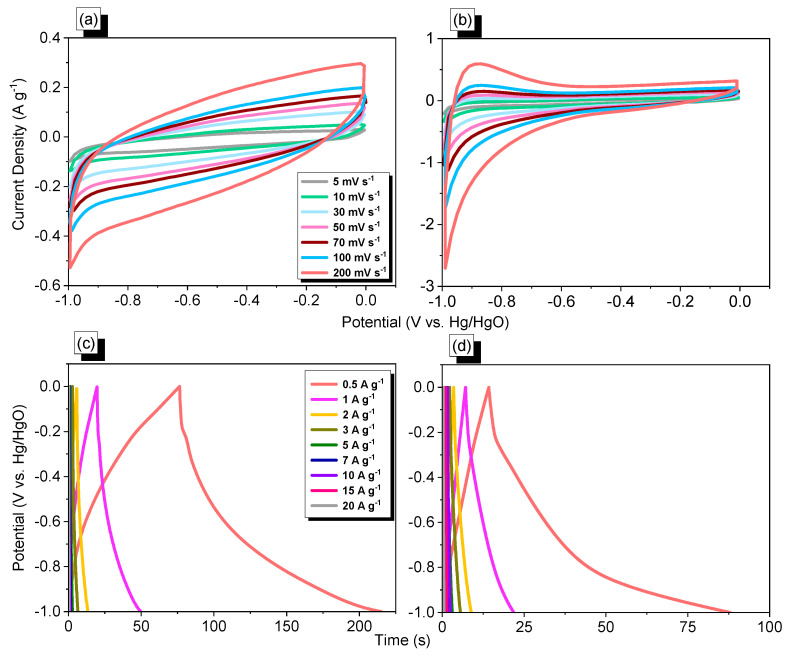
CV (**a**,**b**) and GCD (**c**,**d**) curves of TBN-BSU CMP (**a**,**c**) and Py-BSU CMP (**b**,**d**).

We used 1 M KOH aqueous solution as the electrolyte in a three-electrode system and compared the electrochemical performance of Py-BSU CMP and TBN-BSU CMP by cyclic voltammetry (CV) and galvanostatic charge–discharge (GCD). The working electrode, counter electrode, and reference electrode of the three-electrode system are composed of glassy carbon, platinum, and Hg/HgO, respectively. The CV curves of Py-BSU CMP and TBN-BSU CMP at scan rates from 5 to 200 mV s^−1^ are shown in Figure 5a,b, and the overall leaf-like shape suggests a response originating from the electric double-layer capacitance (EDLC). Among them, as the scan rate increased from 5 to 200 mV s^−1^, the maintained shape of the CV curves indicates the excellent rate capability of the material. Figure 5c,d report the GCD curves detected at current densities ranging from 0.5 to 20 A g^−1^. Slightly curved triangles are characteristic of EDLC.

**Figure 6:**
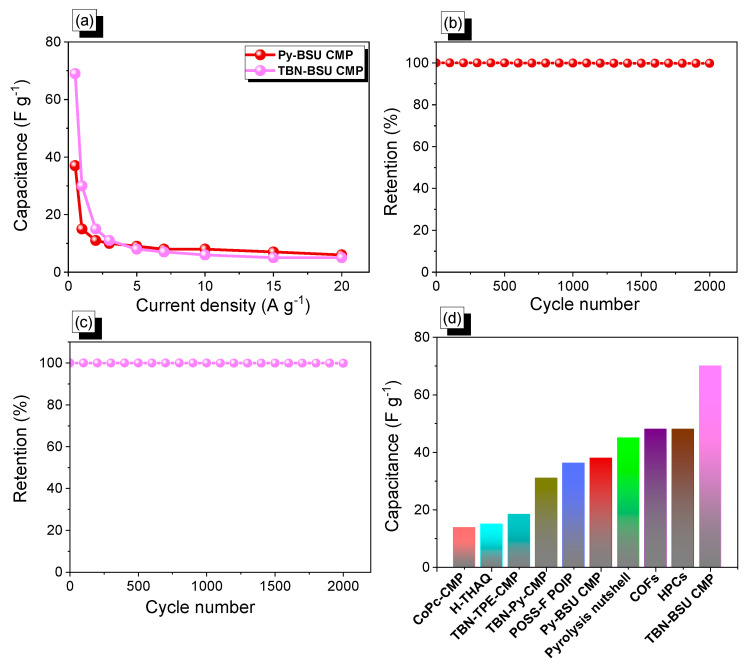
Capacitance (**a**) and stability (**b**,**c**) and the performance of Py-BSU CMP and TBN-BSU CMP precursors compared with other POPs in energy storage (**d**).

As shown in Figure 6a, at a current density of 0.5 A g^−1^, the specific capacitance values of Py-BSU CMP and TBN-BSU CMP calculated by GCD curves were 38 and 70 F g^−1^. In contrast, TBN-BSU CMP exhibited superior electrochemical behavior due to the more extended conjugated structure of the TBN unit and high surface area. The improvement of the π system increased the charge transfer efficiency in contact with the electrolyte and further promoted the performance of the supercapacitor through this process. From another point of view, the capacitance value shrunk when the current density increased from 0.5 to 20 A g^−1^. This can be attributed to the diffusion problem caused by electrolyte ions not fully permeating into the electrode material due to insufficient time in the high current environment. Figure 6b,c demonstrate the measured stability over 2000 cycles at a current density of 10 A g^−1^. The capacitance retention rates of Py-BSU CMP and TBN-BSU CMP were 99.8% and 99.9%, respectively. The extremely small capacitance decay indicates that the material has excellent electrochemical reversibility. Figure 6d shows that the specific capacitance value of TBN-BSU CMP precursor (70 F g^−1^) is higher than CoPc-CMP (13.8 F g^−1^) [78], H-THAQ (15 F g^−1^) [79], TBN-TPE-CMP (18.45 F g^−1^) [32], TBN-Py-CMP (31 F g^−1^) [32], POSS-F-POIP (36.2 F g^−1^) [31], Py-BSU CMP (38 F g^−1^), pyrolysis nutshell (45 F g^−1^) [80], COFs (48 F g^−1^) [81], HPCs (48 F g^−1^) [82], TBN-BSU CMP (70 F g^−1^), and other candidate materials.

**Figure 7:**
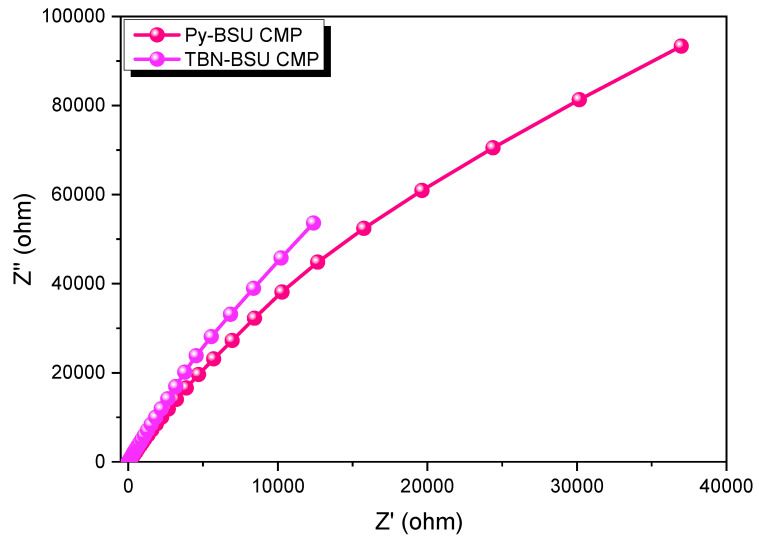
Electrochemical impedance spectroscopy (EIS) of Py-BSU CMP and TBN-BSU CMP electrode materials, recorded at 0.5 Hz.

In Figure 7 and Figure S32, the EIS results indicated that Py-BSU CMP and TBN-BSU CMP have slightly different Nyquist curves, suggesting they have different resistances. In detail, TBN and Py-BSU CMP showed a specific resistance of 9 and 23 ohm, respectively. The charge transfer rate studies were tested through EIS within the frequency range of 100 mHz to 100 kHz at the open circuit potential and 5 mV as an amplitude. It should be noticed that a smaller semi-circuit was observed for TBN-BSU CMP with lower resistance than Py-BSU CMP, indicating a lower resistance and lower charge transfer resistance. The results were fitted to a simulation circuit composed of four elements of the series resistance (R_s_), the charge-transfer resistance (R_ct_), the electrical double-layer capacitance (C_dl_), and the Warburg diffusion impedance (W_d_), as displayed in the inset of the figure of Randles Cell. It is important to notice that low-resistance materials can improve energy storage because they have fast charge transport capabilities. Therefore, TBN-BSU CMP is a better choice for energy storage applications. Due to its high surface area, structural integrity, and compatibility with bis-sulfone units, TBN-BSU CMP exhibited this behavior [83,84,85,86,87,88].

## 3. Materials and Methods

### 3.1. Materials

Anhydrous magnesium sulfate (MgSO_4_, 99.5%), pyrene (Py), benzophenone (99%), PdCl_2_(PPh_3_)_2_, bromine solution (Br_2_), copper(I) iodide (CuI, 99%), dichloromethane (DCM), glacial acetic acid (AcOH), hydrogen peroxide (H_2_O_2_), potassium carbonate (K_2_CO_3_, 99.9%), Pd(PPh_3_)_4_, thianthrene (THT, 99%), titanium tetrachloride (TiCl_4_, 99.9%), trimethylsilylacetylene (TMSA, 98%), triphenylphosine (PPh_3_, 99%), zinc powder (Zn, 98%) were purchased from Sigma-Aldrich (Gillingham, UK) and Acros Organics (Geel, Belgium).

### 3.2. Synthesis of 2,8-Dibromothianthrene (THT-Br_2_)

Br_2_ solution (6 mL, 0.116 mol) was added dropwise to thianthrene (3.24 g, 0.015 mol) dissolved in AcOH (60 mL). The reactant was heated to 90 °C and agitated for one day. Distilled water (30 mL) was then added to the mixture once it had cooled to room temperature. The resultant precipitate was filtered out and properly washed with a 5% NaHCO_3_ solution and water. The further purification by recrystallization from MeOH/DCM afforded THT-Br_2_ a white solid (Scheme S1). FTIR (Figure S1): 3066 cm^−1^. ^1^H NMR (Figure S2): 7.71, 7.62, 7.31 ppm. ^13^C NMR (Figure S3): 138.00–122.44 ppm.

### 3.3. Synthesis of 2,8-Dibromothianthrene-5,5′,10,10′-Tetraoxide (BSU-Br_2_)

H_2_O_2_ (70 mL, 2.984 mol) was added dropwise to THT-Br_2_ (2.55 g, 0.007 mol), dissolved in glacial acetic acid (55 mL). The reactant was heated to 90 °C and stirred for 1 day. After cooling to room temperature, the resulting precipitate was filtered off and washed thoroughly with 5% NaHCO_3_ solution and H_2_O. Finally, the BSU-Br_2_ product was purified by recrystallization from MeOH/DCM mixture affording BSU-Br_2_ a white solid (Scheme S2). FTIR (Figure S4): 3068 and 1171(SO_2_ group) cm^−1^. ^1^H NMR (Figure S5): 8.38–7.52 ppm (aromatic protons). ^13^C NMR (Figure S6): 137.54–124.62 ppm.

### 3.4. Synthesis of 1,3,6,8-Tetrabromopyrene (Py-Br_4_)

Br_2_ (3.5 mL, 0.068 mol) was injected dropwise into a Py monomer (3.00 g, 0.015 mol) dissolved in nitrobenzene (30 mL). The reaction mixture was then heated to 120 °C and stirred for 24 h. After filtration through EtOH, the green solid was collected and dried at 60 °C (Scheme S3). FTIR (Figure S7): 3053 and 682 (C-Br group) cm^−1^.

### 3.5. Synthesis of 1,3,6,8-Tetrakis(trimethylsilanylethynyl)pyrene (Py-TMS)

Py-Br_4_ (1.00 g, 1.924 mmol), Pd(PPh_3_)_4_ (0.13 g, 0.112 mmol), PPh_3_ (0.11 g, 0.419 mmol), and CuI (0.06 g, 0.315 mmol) were dissolved in Et_3_N (28 mL) and anhydrous toluene (28 mL). TMSA (1.52 g, 15.48 mmol) was added dropwise to the reactant after heating to 50 °C. The mixture was then heated to 80 °C for two days. The solvent was removed via a rotary evaporator, and the orange solid dried under vacuum at 60 °C (Scheme S3). FTIR (KBr, Figure S8): 2908 (aliphatic C-H stretching), 2100 (C≡C stretching). ^1^H NMR (Figure S9): 8.57-8.30 (aromatic), 0.413 (36H). ^13^C NMR (Figure S10): 135.70–101.60.

### 3.6. Synthesis of 1,3,6,8-Tetraethynylpyrene (Py-T)

K_2_CO_3_ (5.70 g, 41.2 mmol) and Py-TMS (2.00 g, 3.395 mmol) were dissolved in anhydrous MeOH (50 mL). The mixture was stirred for two days at room temperature. Filtering and drying the solid at 60 °C were done to afford a brown solid (Scheme S3). FTIR (Figure S11): 3279 (≡C–H), 2186 (C≡C). ^1^H NMR (Figure S12): 8.68–8.38 (aromatic), 3.67 (s, 4H). ^13^C NMR (Figure S13): 133.80–82.00 ppm.

### 3.7. Synthesis of Tetraphenylethylene (TPE), 1,1,2,2-Tetrakis(4-bromophenyl)ethene (TPE-Br_4_) and 2,7,10,15-Tetrabromodibenzo[g,p]chrysene (TBN-Br_4_)

The synthesis detail for TPE, TPE-Br_4_, and TBN-Br_4_ are provided in the supporting information file, and their FTIR and NMR data confirmed their chemical structure (Schemes S4 and S5, Figures S14–S22).

### 3.8. Synthesis of 2,7,10,15-Tetrakis(trimethylsilyl)ethynyl)dibenzo[g,p]chrysene (TBN-TMS)

In 125 mL of diethylamine, 1.5 g of TBN-Br_4_ (2.33 mmol) was dissolved. Then the solution was treated with a combination of CuI (0.11 g), PdCl_2_(PPh_3_)_2_ (0.08 g), and ethynyltrimethylsilane (1.83 g, 18.63 mmol). After 24 h of stirring at 80 °C, the solvent was removed under reduced pressure, and the product was then purified using flash chromatography on a silica gel column with DCM as an eluent to produce a yellow solid (Scheme S6, 1.3 g, 86%). FTIR (Figure S23): 3291 (C≡C-H), 3031 (aromatic C-H), 2957 (aliphatic C-H), 2156 (C≡C), 1632 (C=C). ^1^H NMR (Figure S24): δ 8.77, 8.47, 7.69, 0.34 (s, 36H, CH_3_). ^13^C NMR (Figure S25): 131.24, 128.74, 121.90, 105.38, 96.34, 30.59, 3.50.

### 3.9. Synthesis of 2,7,10,15-Tetraethynyldibenzo[g,p]chrysene (TBN-T)

The TBN-TMS (1 g, 1.40 mmol) was dissolved in 60 mL of MeOH and 40 mL of DCM before being mixed for 24 h with 1.2 g of K_2_CO_3_ (8.65 mmol). Following this, the solvent was removed using a vacuum. The resulting solid was dissolved with 50 mL of DCM and then extracted with H_2_O to afford TBN-T (0.8 g, 80%) as an orange solid after the solvent was removed under vacuum (Scheme S7). FTIR (KBr, cm^−1^, Figure S26): 3291 (C≡C-H), 3057 (aromatic C-H), 2106 (C≡C), 1604 (C=C). ^1^H NMR (Figure S27): 8.81 (s, 4H), 8.53 (s, 4H), 7.73 (s, 4H), 3.30 (s, 4H). ^3^C NMR (Figure S28): 135.70, 132.40, 127.80, 119.20, 103.50, 101.60, 84.21, 78.87.

### 3.10. Synthesis of Py-BSU CMP

BSU-Br_2_ (0.293 g, 0.669 mmol), Py-T (0.1 g, 0.333 mmol), Pd(PPh_3_)_4_ (0.038 g, 0.033 mmol), PPh_3_ (0.0087 g, 0.033 mmol), and CuI (0.0063 g, 0.033 mmol) were added to Et_3_N (7 mL) and DMF (7 mL) and heated to 90 °C for 3 days. The filtered powder was washed stepwise with THF, MeOH, and acetone. The solid was dried under vacuum at 110 °C overnight to obtain a dark red powder (Scheme 1a).

### 3.11. Synthesis of TBN-BSU CMP

BSU-Br_2_ (0.206 g, 0.470 mmol), TBN-T (0.1 g, 0.236 mmol), Pd(PPh_3_)_4_ (0.027 g, 0.023 mmol), PPh_3_ (0.006 g, 0.023 mmol), and CuI (0.006 g, 0.032 mmol) was added in Et_3_N (5 mL) and DMF (5 mL) and heated to 90 °C for 3 days. The filtered powder was washed well step by step with THF, MeOH, and acetone. Dried the solid under vacuum at 110 °C overnight and then gained a yellow powder (Scheme 1b).

## 4. Conclusions

In this study, we successfully synthesized two conjugated microporous polymers, Py-BSU CMP and TBN-BSU CMP, using Sonogashira-Hagihara cross-coupling of BSU-Br_2_ with Py-T and TBN-T, respectively. We characterized the chemical structures, physical properties, porosity, and morphology of these CMPs using various analytical techniques, including FTIR, solid-state ^13^C NMR spectroscopy, BET, SEM, and TEM. Both Py-BSU CMP and TBN-BSU CMP showed excellent thermal stability with T_d10_ up to 383 °C and char yield up to 48 wt% at 800 °C as measured by TGA. We also investigated the electrochemical performance of these CMPs and found that TBN-BSU CMP exhibited better performance due to its more extended π-conjugated system, higher specific surface area (391 m^2^ g^−1^), and total pore volume (0.30 cm^3^ g^−1^). Under the three-electrode measurement system, TBN-BSU CMP showed significantly better electrochemical performance with a capacitance of 70 F g^−1^ at a current density of 0.5 A g^−1^ compared to Py-BSU CMP. TBN-BSU CMP also demonstrated excellent stability with less capacitance decay (99.9%) in long-life-cycle energy storage devices. Finally, we believe that these BSU-linked CMPs, Py-BSU and TBN-BSU CMPs, hold great potential for other applications such as H_2_ production and gas conversion. Overall, our findings suggest that these CMPs could be attractive candidates for various energy storage and conversion applications.

## Figures and Tables

**Table 1:** Thermal stabilities and porosity properties of Py-BSU CMP and TBN-BSU CMP.

Sample	T_d5_ (°C)	T_d10_ (°C)	Char Yield (wt.%)	S_BET_ (m^2^ g^−1^)	Pore Volume (cm^3^ g^−1^)	Pore Size (nm)
Py-BSU CMP	320	383	48	42	0.07	2.57
TBN-BSU CMP	338	386	56	391	0.30	1.80

## Data Availability

The data presented in this study are available on request from the corresponding author.

## Supplementary Materials

The following supporting information can be downloaded at: https://www.mdpi.com/article/10.3390/molecules28073234/s1, Scheme S1: Synthesis of THT-Br_2_; Scheme S2: Synthesis of BSU-Br_2_; Scheme S3: Synthesis of Py-T; Scheme S4: Synthesis of TPE and TPE-Br_4_; Scheme S5: Synthesis of TBN-Br_4_; Scheme S6: Synthesis of TBN-TMS; Scheme S7: Synthesis of TBN-T; Figure S1: FTIR profile of THT-Br_2_; Figure S2: ^1^H-NMR spectrum of THT-Br_2_ in CDCl_3_; Figure S3: ^13^C-NMR spectrum of THT-Br_2_ in CDCl_3_; Figure S4: FTIR profile of BSU-Br_2_; Figure S5: ^1^H-NMR spectrum of BSU-Br_2_; Figure S6: ^13^C-NMR spectrum of BSU-Br_2_; Figure S7: FT-IR spectrum of Py-Br_4_; Figure S8: FT-IR spectrum of Py-TMS; Figure S9: ^1^H NMR spectrum of Py-TMS; Figure S10: ^13^C NMR spectrum of Py-TMS; Figure S11: FT-IR spectrum of Py-T; Figure S12: ^1^H NMR spectrum of Py-T; Figure S13: ^13^C NMR spectrum of Py-T; Figure S14: FT-IR spectrum of TPE; Figure S15: ^1^H NMR spectrum of TPE; Figure S16: ^13^C NMR spectrum of TPE; Figure S17: FT-IR spectrum of TPE-Br_4_; Figure S18: ^1^H NMR spectrum of TPE-Br_4_; Figure S19: ^13^C NMR spectrum of TPE-Br_4_; Figure S20: FTIR spectrum of TBN-Br_4_; Figure S21: ^1^H-NMR spectrum of TBN-Br_4_. Figure S22. ^13^C-NMR spectrum of TBN-Br_4_. Figure S23. FTIR spectrum of TBN-TMS; Figure S24: ^1^H NMR spectrum of TBN-TMS; Figure S25: ^13^C NMR spectrum of TBN-TMS; Figure S26: FTIR spectrum of TBN-T; Figure S27: ^1^H NMR spectrum of TBN-T; Figure S28: ^13^C NMR spectrum of TBN-T; Figure S29: XRD profiles of Py-BSU CMP and TBN-BSU CMP; Figure S30: Weight elements contents profiles of (a) Py-BSU CMP and (b) TBN-BSU CMP; Figure S31: TEM images of Py-BSU CMP and TBN-BSU CMP; Figure S32: Fitted circuits of the Nyquist plots of Py-BSU CMP and TBN-BSU CMP.
