# Supplementary material for: Design and Synthesis of Bisulfone-Linked Two-Dimensional Conjugated Microporous Polymers for CO2 Adsorption and Energy Storage
Source: Molecules. 2023 Apr 4;28(7):3234. doi: 10.3390/molecules28073234 (PMC10096630; doi:10.3390/molecules28073234)
Supplement: Supplementary file 1 [file molecules-28-03234-s001.zip › molecules-2308736-supplementary.pdf]

Supporting Information

Design and Synthesis of Bisulfone Linked Two-Dimensional Conjugated Microporous Polymers for CO_2 adsorption and Energy Storage

Mohamed Gamal Mohamed[1,2]*, Siang-Yi Chang[1], Moshin Ejaz[1], Maha Mohamed Samy[1], Aya Osama Mousa[1] and Shiao-Wei Kuo[1,3]*

[1]Department of Materials and Optoelectronic Science, College of Semiconductor and Advanced Technology Research, Center of Crystal Research, National Sun Yat-Sen University, Kaohsiung 804, Taiwan.

[2]Chemistry Department, Faculty of Science, Assiut University, Assiut 71515, Egypt.

[3]Department of Medicinal and Applied Chemistry, Kaohsiung Medical University, Kaohsiung 807, Taiwan.

Corresponding authors:

E-mail: mgamal.eldin34@gmail.com (M. G. M) and kuosw@faculty.nsysu.edu.tw (S. W. K).

Characterization

FTIR spectra were recorded using a Bruker Tensor 27 FTIR spectrophotometer with a resolution of 4 cm^{-1} through the KBr disk method. ^{1}H and ^{13}C Nuclear magnetic resonance (NMR) spectra were recorded using an INOVA 500 instrument with $CDCl_3$ as the solvent and TMS as the external standard; chemical shifts are reported in parts per million (ppm). Dynamic thermal curing kinetics were measured using a TA Q-20 differential scanning calorimeter with ca. 7 mg sample on the DSC sample pan under a N_2 atmosphere (100 mL min^{-1}), with heating from -80 to 350 °C at a heating rate of 10 °C min^{-1}. The thermal stabilities of the samples were examined under a N_2 using a TA Q-50 thermogravimetric analyzer; each cured sample (ca. 5 mg) was placed in a Pt cell and heated at a rate of 20 °C min^{-1} from 40 to 800 °C under a N_2 flow rate of 60 mL min^{-1}. The morphologies of the polymer samples were examined using scanning electron microscopy (SEM; JEOL-6330) and using transmission electron microscopy (TEM; JEOL-2100) instrument operated at an accelerating voltage of 200 kV.

Electrochemical Analysis

Working Electrode Cleaning: Prior to using, the glassy carbon electrode (GCE) was polished several times with 0.05-μm alumina powder, washed with EtOH after each polishing step, cleaned through sonication (5 min) in a water bath, washed with EtOH, and then dried in the oven.

Electrochemical Characterization: The electrochemical experiments were performed in a three-electrode cell using an Autolab potentiostat (PGSTAT204) and 1 M KOH as the aqueous electrolyte. The GCE was used as the working electrode (diameter: 5.61 mm; 0.2475 cm^2); a Pt wire was used as the counter electrode; Hg/HgO (RE-1B, BAS)

was the reference electrode. All reported potentials refer to the Hg/HgO potential. A slurry was prepared by dispersing the Py-BSU and TBN-BSU CMP sample (45 wt %), carbon black (45 wt %), and Nafion (10 wt %) in a mixture of (EtOH/ H_2O) (200 μL: 800 μL) and then sonicated for 1 h. A portion of this slurry (10 μL) was pipetted onto the tip of the electrode, which was then dried in the oven for 30 min. The electrochemical performance was studied through CV at various sweep rates (5–200 mV s^{-1}) and through the GCD method in the potential range from -1.0 V and 0.0 V (vs. Hg/HgO) at various current densities (0.5–20 A g^{-1}) in 1 M KOH as the aqueous electrolyte solution.

The specific capacitance was calculated from the GCD data using the equation.

$$C_s = (I\Delta t)/(m\Delta V) \quad \textbf{(1)}$$

Where C_s (F g^{-1}) is the specific capacitance of the supercapacitor, I (A) is the discharge current, ΔV (V) is the potential window, Δt (s) is the discharge time, and m (g) is the mass of the NPC on the electrode. The energy density (E, W h kg^{-1}) and power density (P, W kg^{-1}) were calculated using the equations.

$$E = 1000C(\Delta V)^2/(2 \times 3600) \quad \textbf{(2)}$$

$$P = E/(t/3600) \quad \textbf{(3)}$$

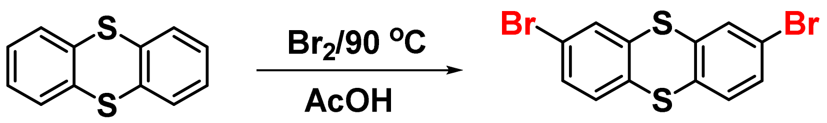

Scheme S1. Synthesis of THT-Br_2.

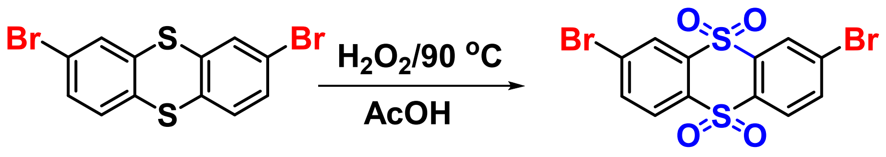

Scheme S2. Synthesis of BSU-Br_2.

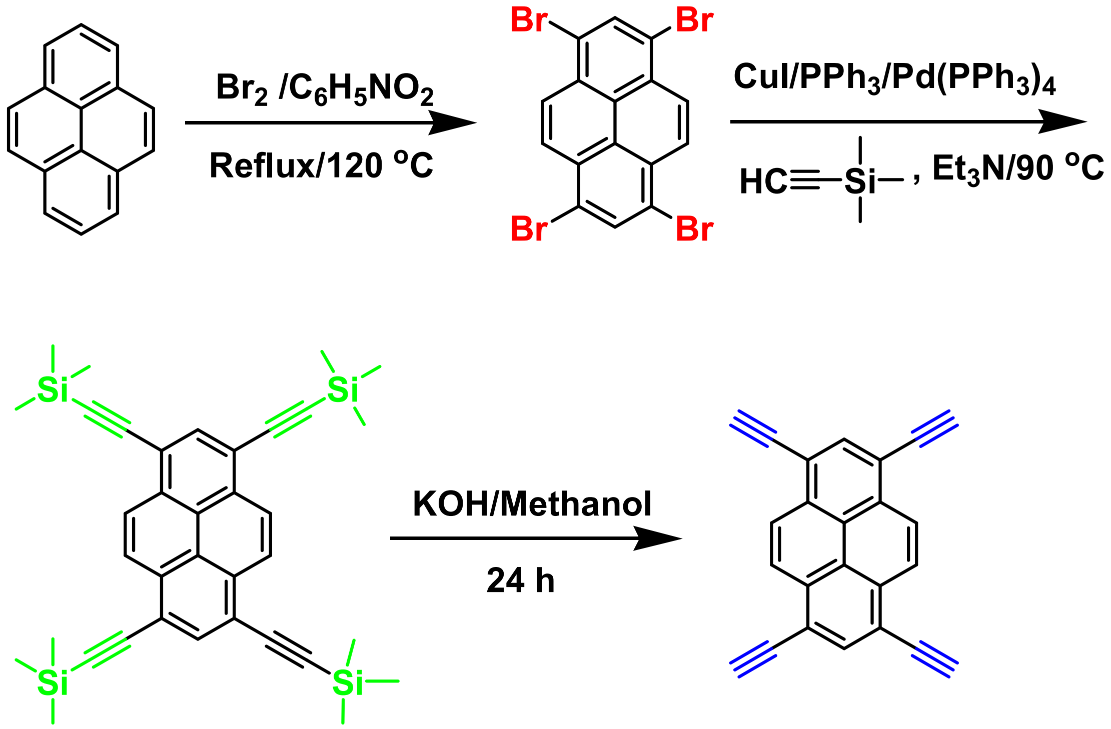

Scheme S3. Synthesis of Py-T.

Synthesis of Tetraphenylethylene (TPE) and 1,1,2,2-Tetrakis(4-bromophenyl)ethene (TPE-Br_4)

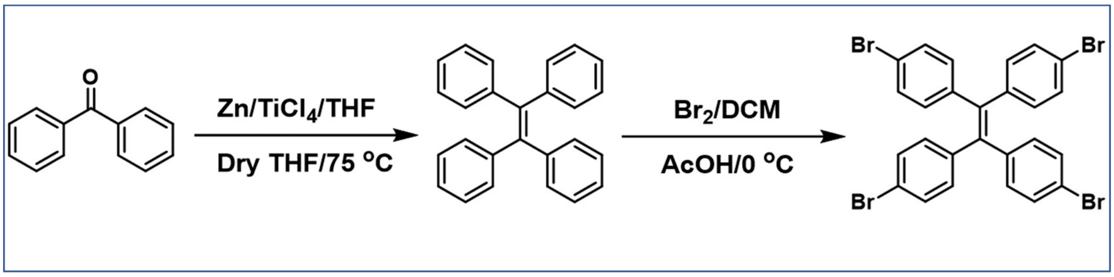

Scheme S4. Synthesis of TPE and TPE-Br_4.

under N_2, benzophenone (3.00 g, 16.4 mmol) and Zn (4.31g, 65.9 mmol) in THF (80 mL) were stirred in an ice/salt-water bath for 10 min. $TiCl_4$ (3.60 mL, 33.0 mmol) was injected over 30 min, and then the mixture was kept at 80 °C under reflux. Then, 5% aqueous K_2CO_3 was added to the reaction. After evaporation of the organic solvent, EtOAc extracted the aqueous phase three times. After evaporation of EtOAc, the residue was washed with EtOH to obtain a white crystalline solid (2.66 g, 97%). M.p.: 228–229 °C (DSC). FTIR (KBr, cm^{-1}, **Figure S14**): 3047 (aromatic C–H stretching), 1602 (C=C stretching). ^1H NMR (500 MHz, $CDCl_3$, δ, ppm, **Figure S15**): 7.26 (d, 8H), 6.84 (d, 8H). ^{13}C NMR (125 MHz, $CDCl_3$, δ, ppm, **Figure S16**): 140.70, 141.00, 131.30, 127.70, 126.4. A solution of TPE (3.32 g, 10.0 mmol) in glacial acetic acid (10 mL) and CH_2Cl_2 (20 mL) in a round-bottom flask at 0 °C (ice bath). Br_2 (4.00 mL, 80.0 mmol) was added to the mixture and kept at room temperature for 48 h. Then, the H_2O (200 mL) was added to the resulting solution, and the mixture was extracted with CH_2Cl_2. After evaporation of CH_2Cl_2, the residue was washed with MeOH to give a white solid, which was recrystallized (CH_2Cl_2/MeOH) to give TPE-Br_4 a white crystalline solid (6.15 g, 95%). M.p.: 261–262 °C (DSC). FTIR (KBr, cm^{-1}, **Figure S17**): 3051 (aromatic C–H stretching), 1572 (C=C stretching). ^1H NMR (500 MHz, $CDCl_3$, δ, ppm, **Figure S18**): 7.25 (d, 8H), 6.84 (d, 8H). ^{13}C NMR (125 MHz, $CDCl_3$, δ, ppm, **Figure S19**): 142.30, 139.70, 133.70, 131.90, 121.80.

Synthesis of 2,7,10,15-tetrabromodibenzo[g,p]chrysene (TBN-Br$_4$)

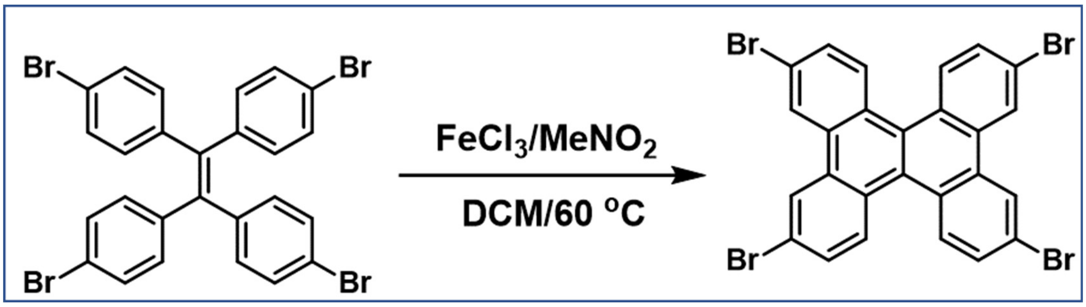

Scheme S5. Synthesis of TBN-Br$_4$.

TPE-Br$_4$ (1.0 g, 1.54 mmol) was subsequently mixed in dry DCM (40 mL) under the presence of a nitrogen atmosphere. Following that, a septum was used to add solutions of $FeCl_3$ (4.54 g, 27.2 mmol) in dry nitromethane (10 mL) to the reaction mixture. The materials were warmed at reflux for approximately 4 h before being cooled to room temperature. The reaction was stopped by adding 50 mL of methanol. The chemical components were then extracted with $CHCl_3$, washed with a diluted $NaHCO_3$ solution, and evaporated until dryness to obtain TBN-Br$_4$ as a yellow solid (0.71 g, 76%); FTIR (KBr, cm^{-1}, **Figure S20**): 3078 (aromatic C-H), 1594 (C=C), 591. ^{1}H NMR (500 MHz, $CDCl_3$, **Figure S21**): 7.77 (s, 4H), 8.42 (s, 4H), 8.73 (s, 4H). ^{13}C NMR (500 MHz, $CDCl_3$, **Figure S22**): 135.70, 132.40, 127.80, 119.20, 103.50, 101.60.

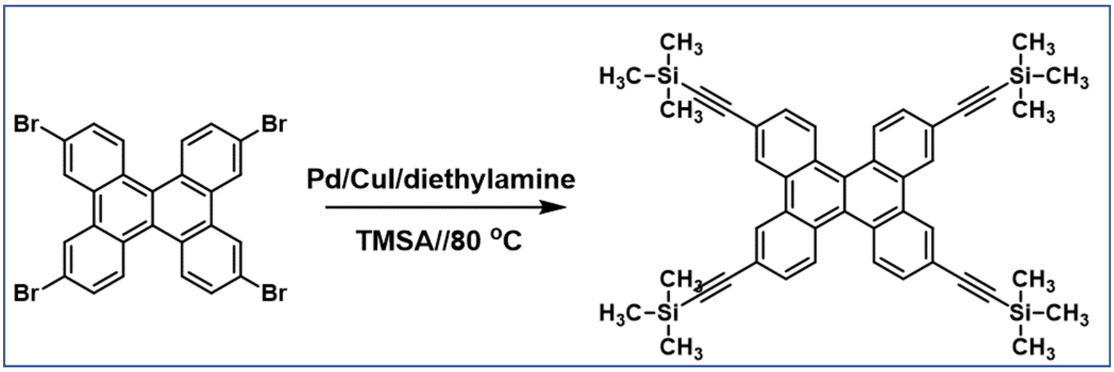

Scheme S6. Synthesis of TBN-TMS.

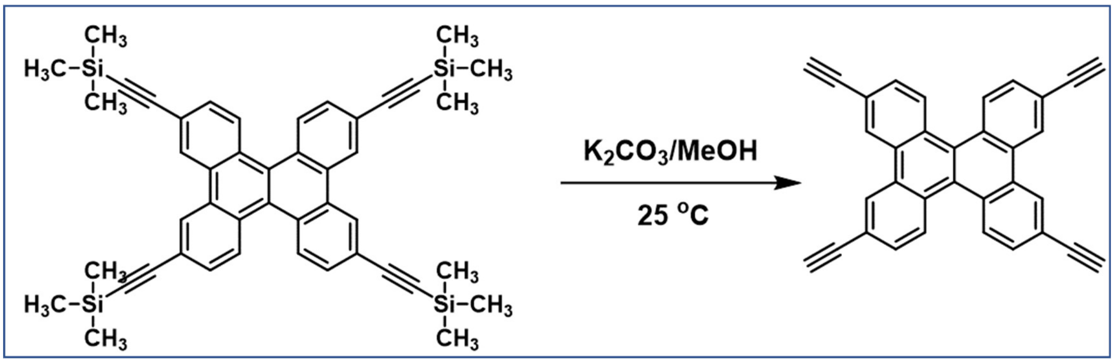

Scheme S7. Synthesis of TBN-T.

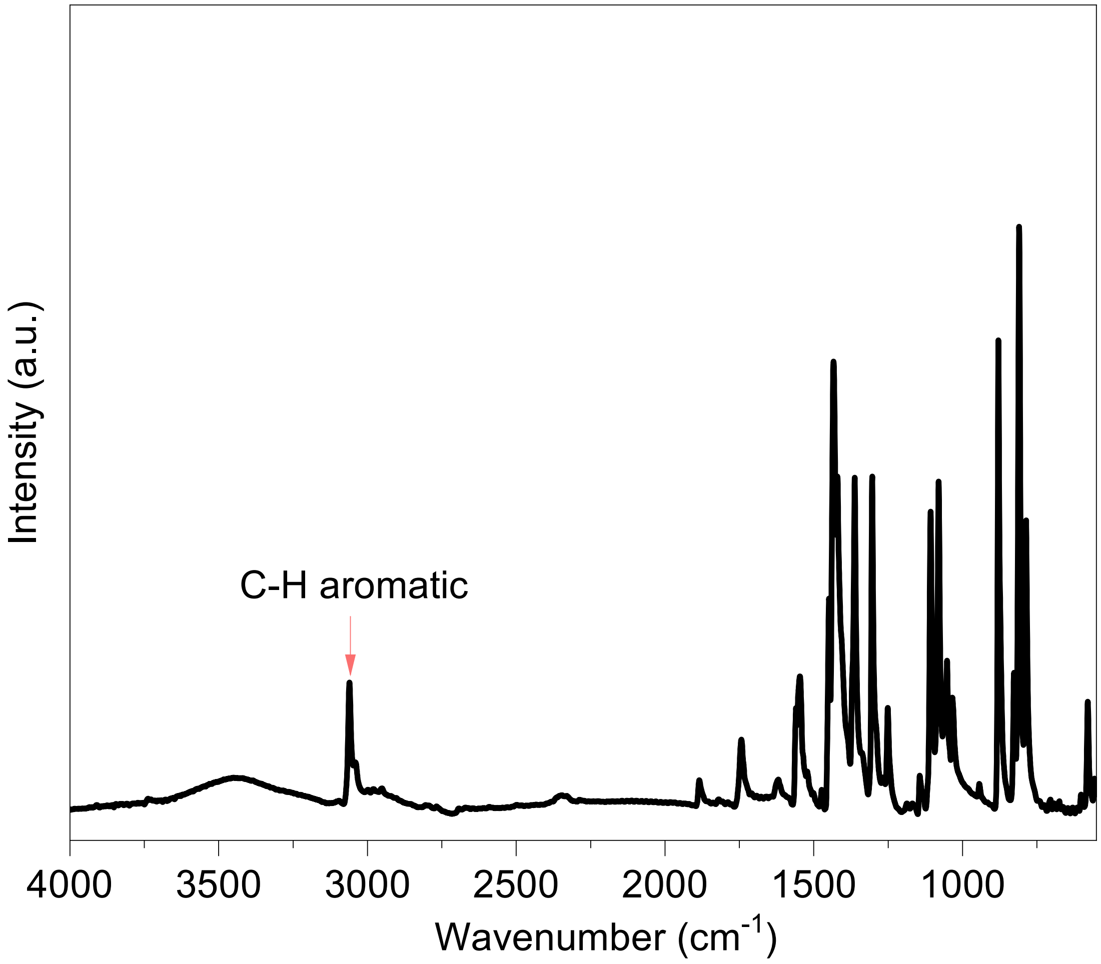

Figure S1. FTIR profile of THT-Br_2.

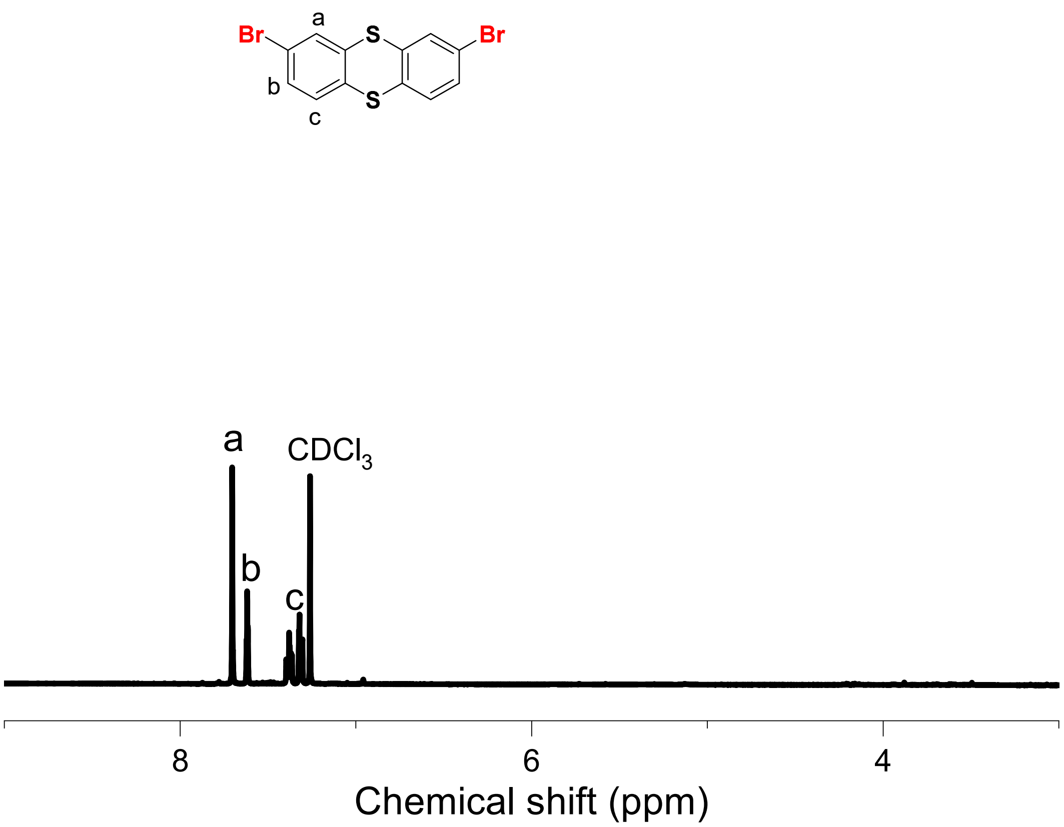

Figure S2. ^{1}H-NMR spectrum of THT-Br_2 in $CDCl_3$.

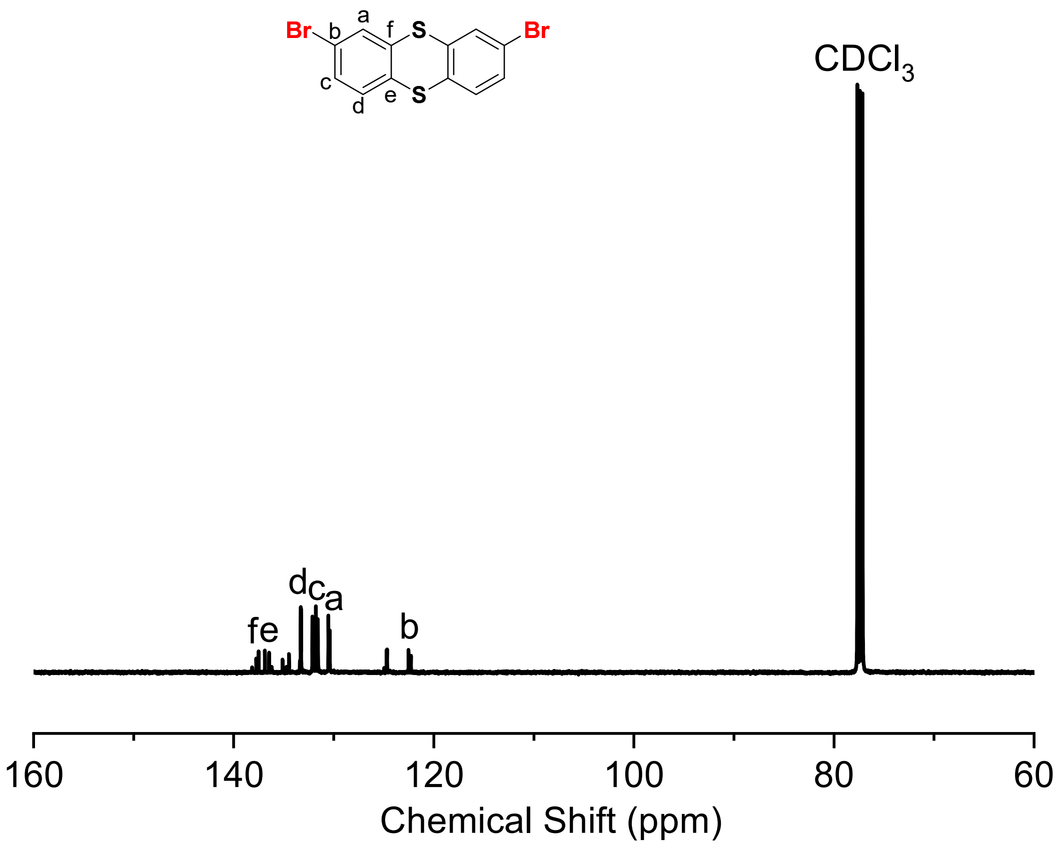

Figure S3. ^{13}C-NMR spectrum of THT-Br_2 in $CDCl_3$.

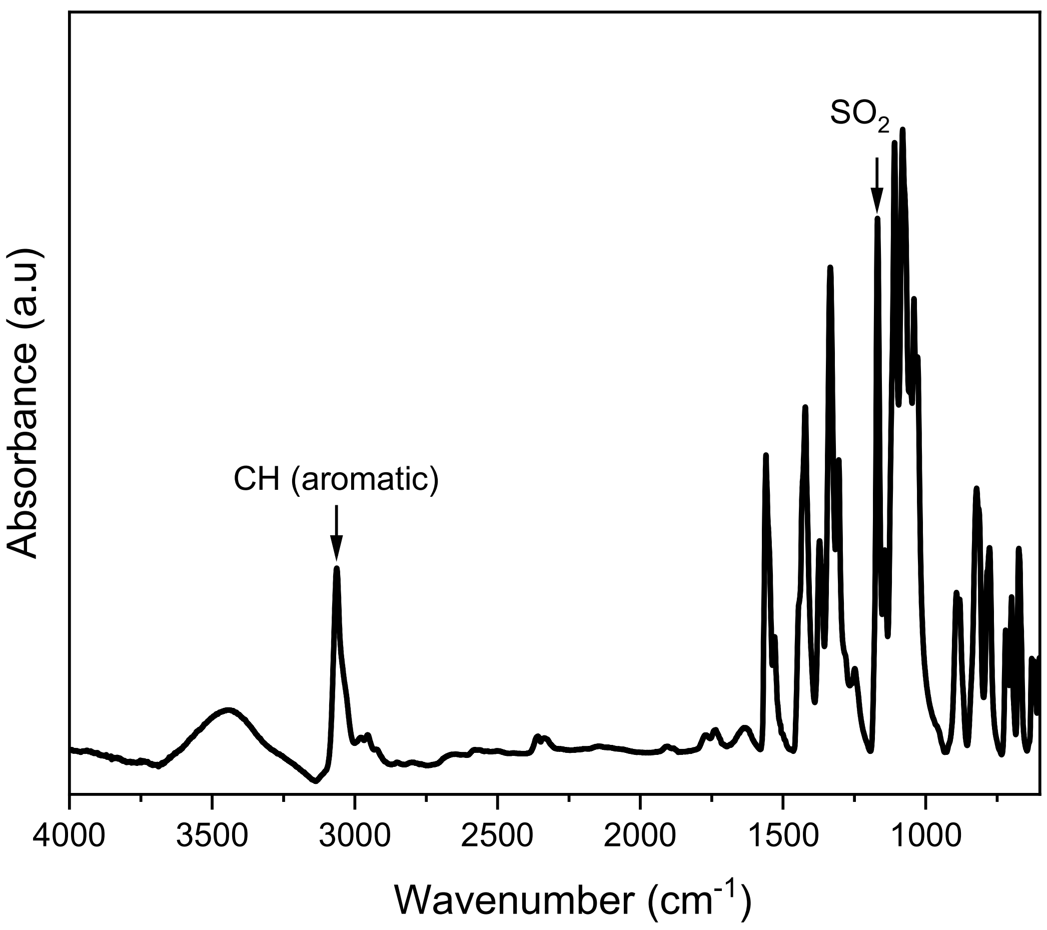

Figure S4. FTIR profile of BSU-Br_2.

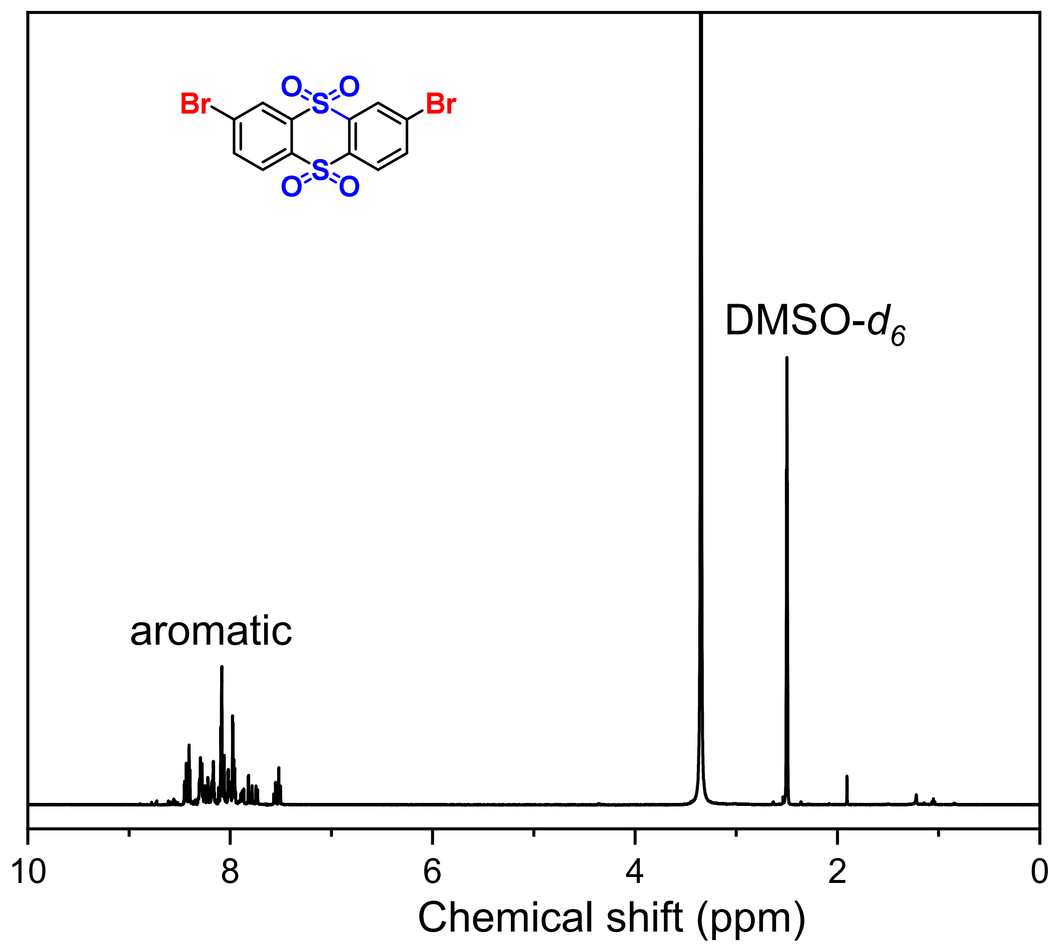

Figure S5. ^{1}H-NMR spectrum of BSU-Br_2.

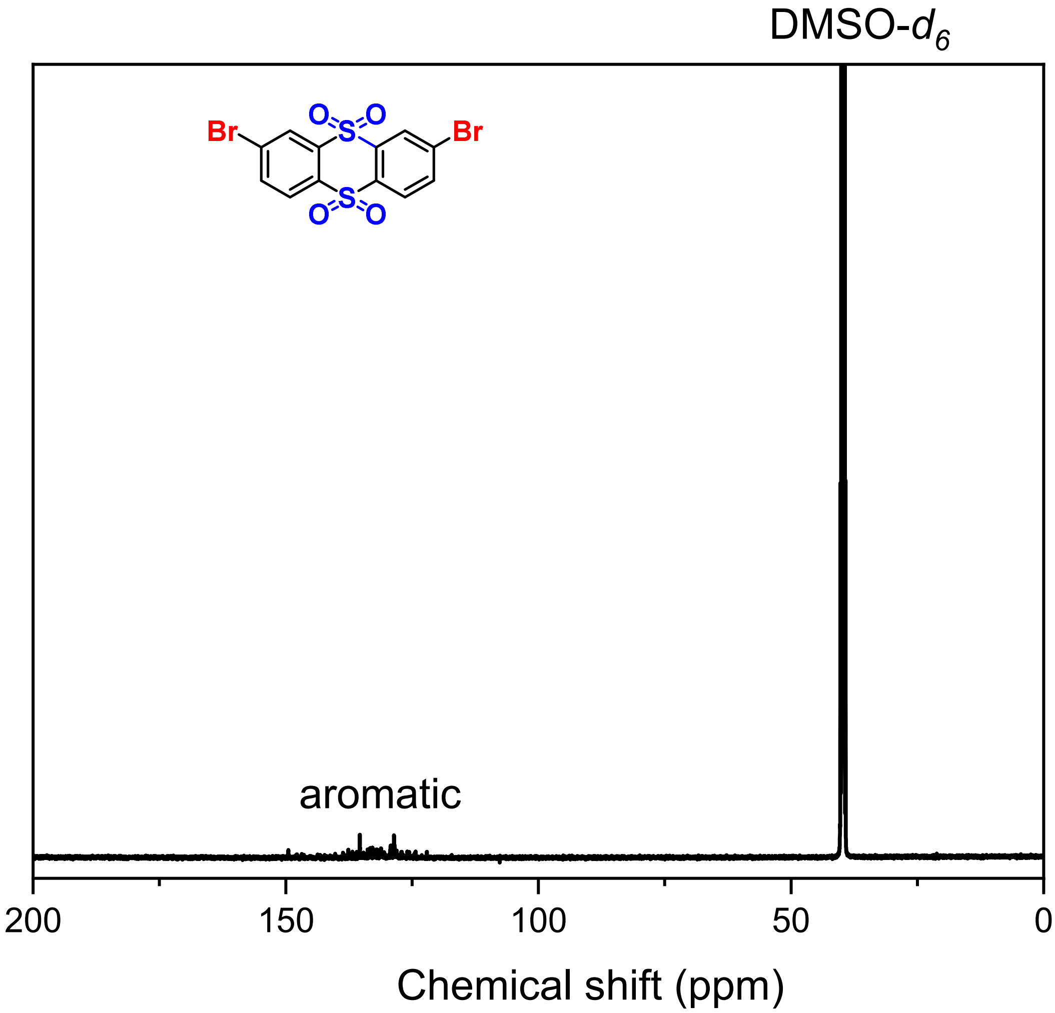

Figure S6. ^{13}C-NMR spectrum of BSU-Br_2.

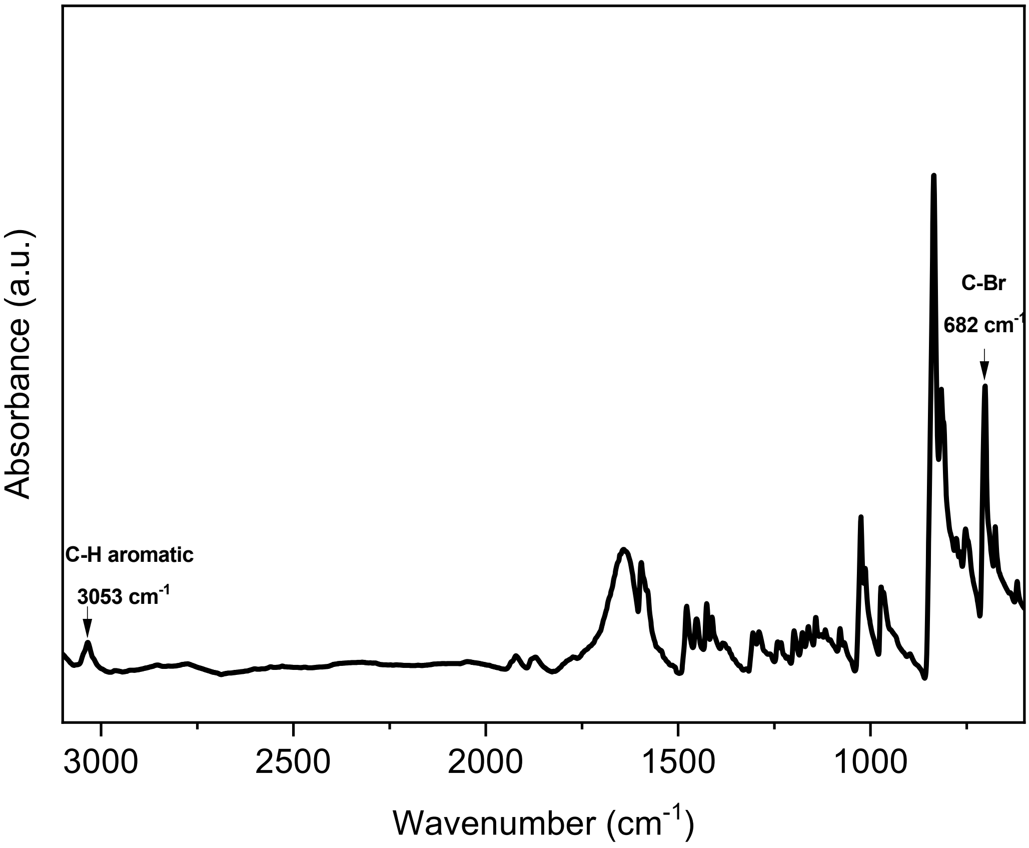

Figure S7. FT-IR spectrum of Py-Br_4.

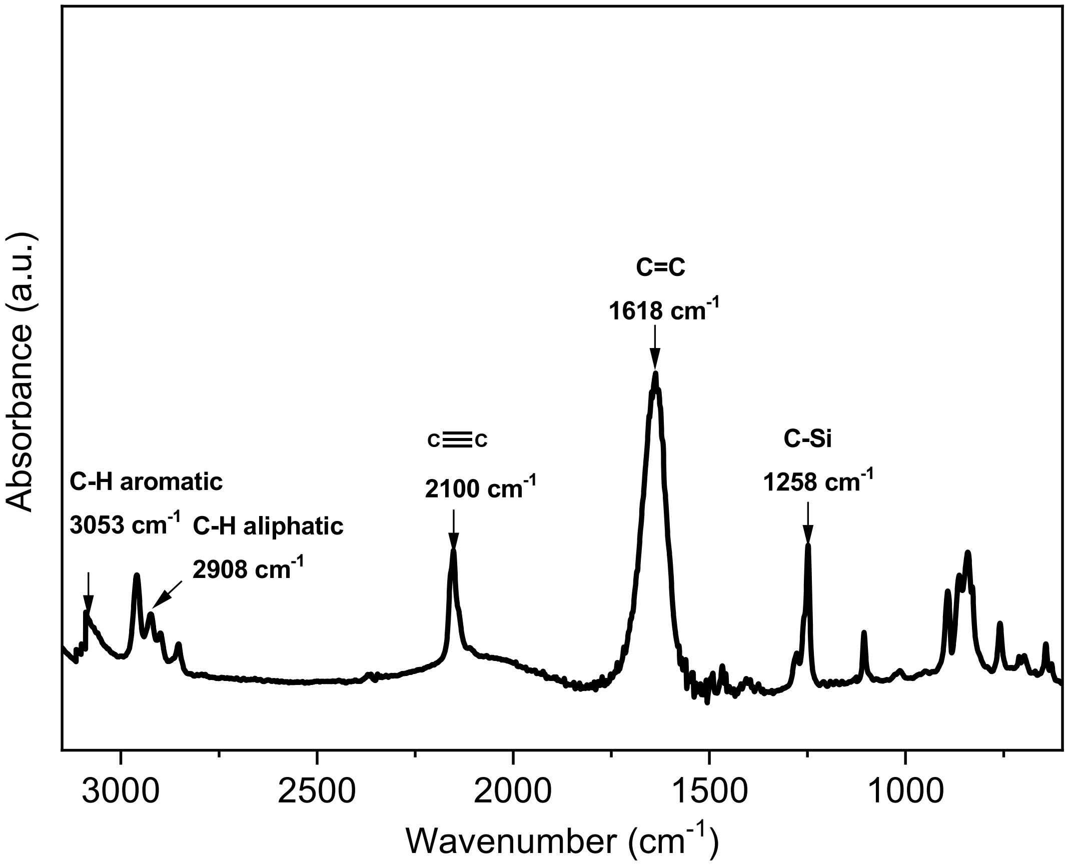

Figure S8. FT-IR spectrum of Py-TMS.

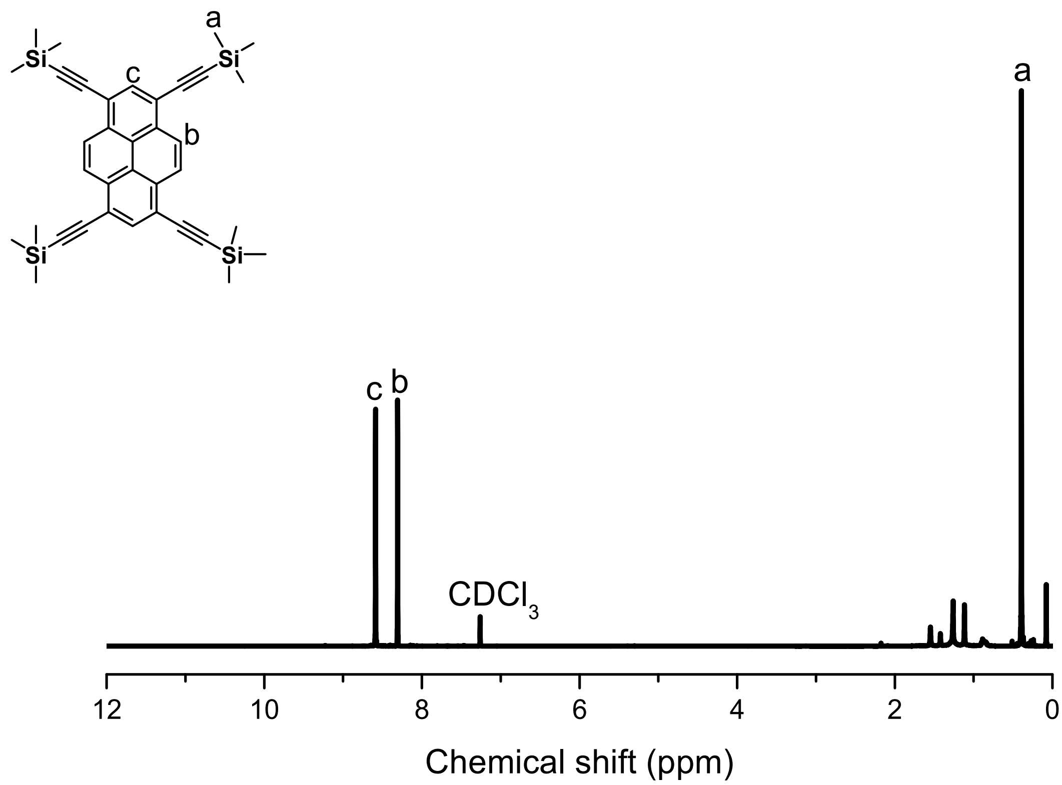

Figure S9. ^{1}H NMR spectrum of Py-TMS.

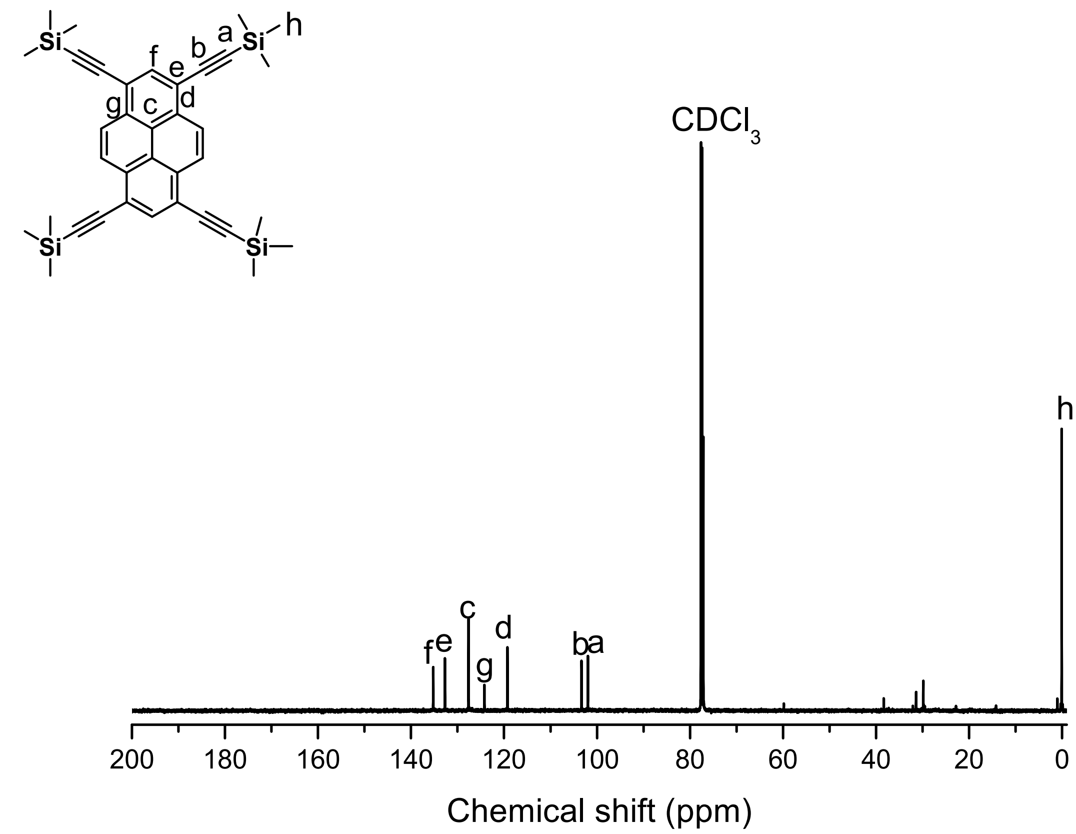

Figure S10. ^{13}C NMR spectrum of Py-TMS.

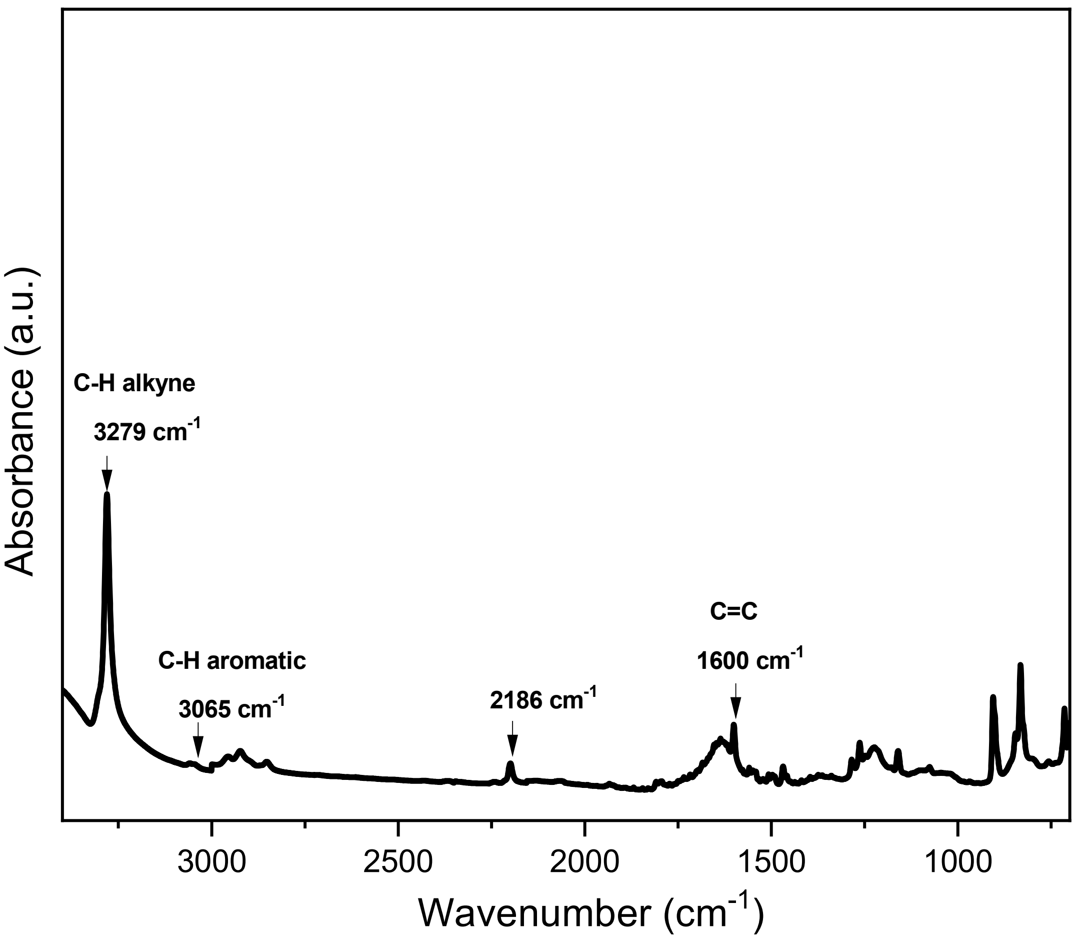

Figure S11. FT-IR spectrum of Py-T.

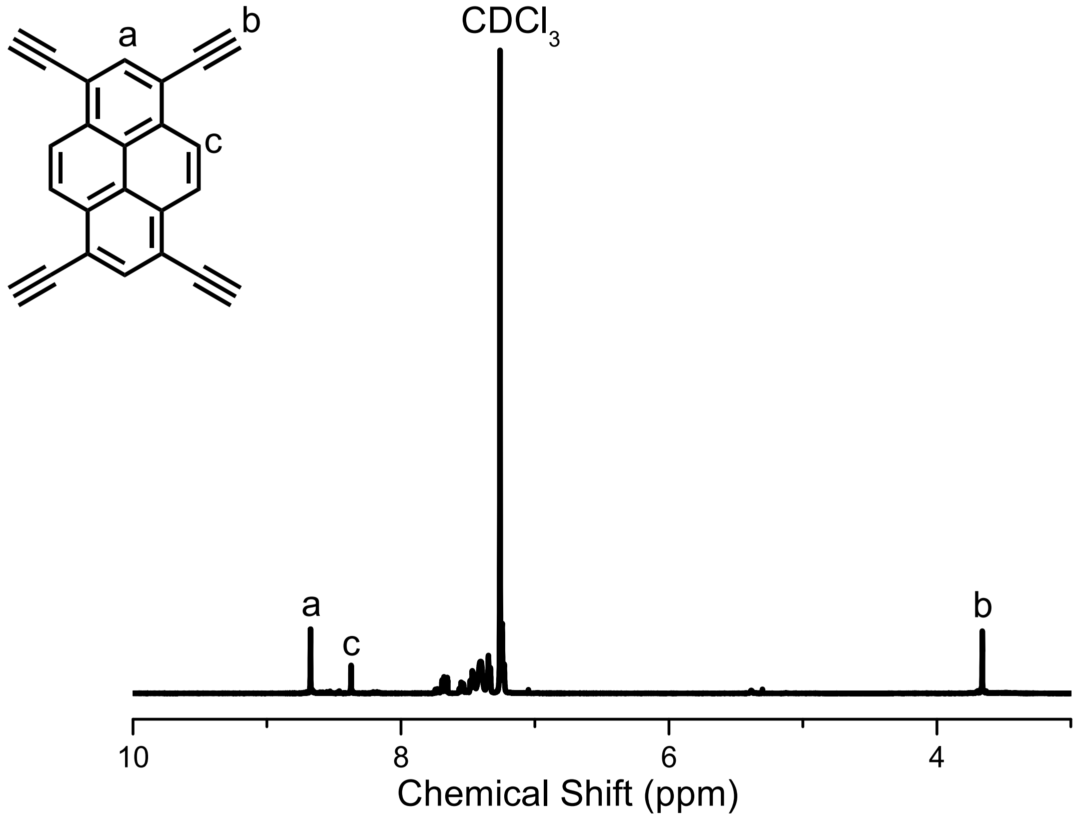

Figure S12. ^{1}H NMR spectrum of Py-T.

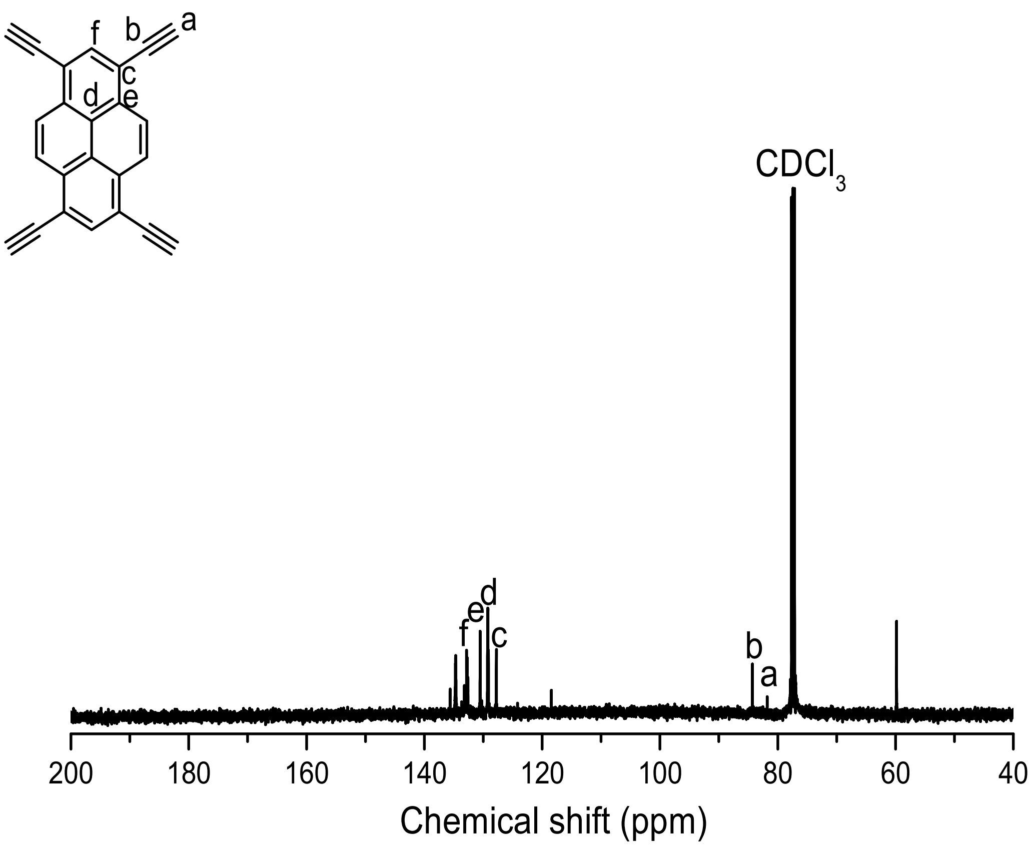

Figure S13. ^{13}C NMR spectrum of Py-T.

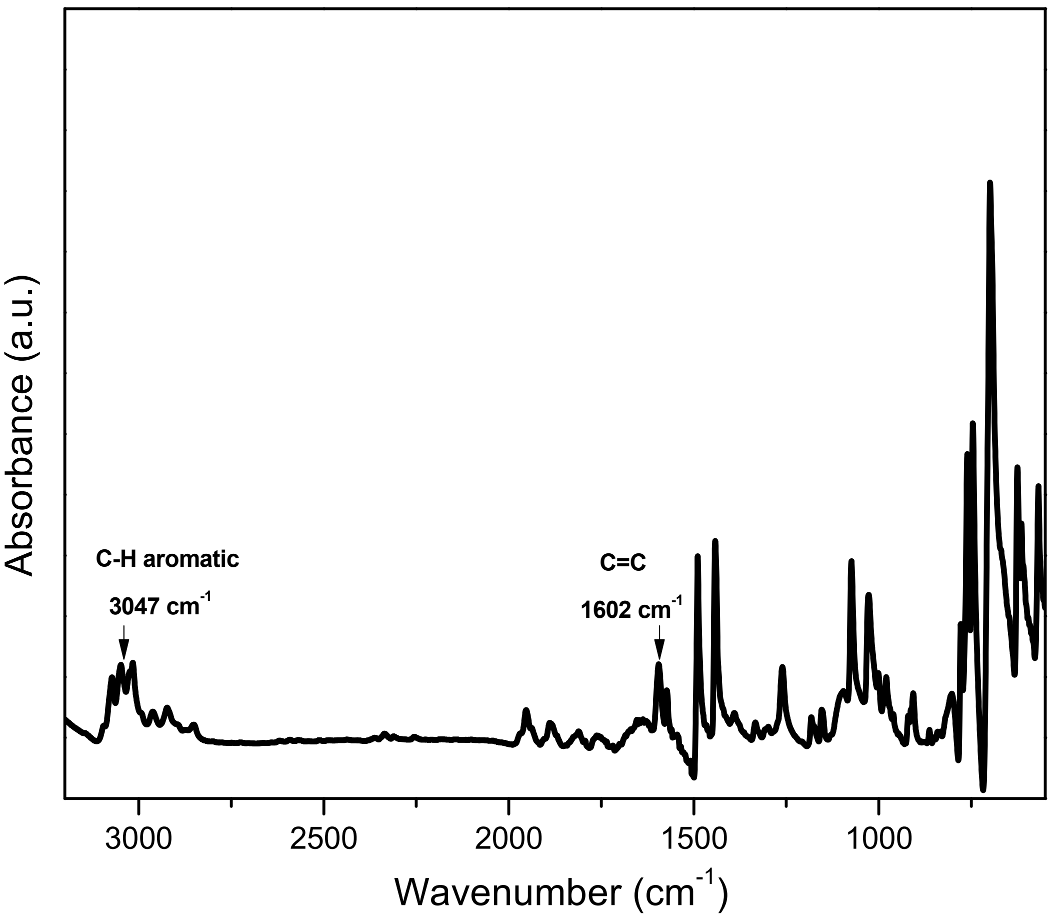

Figure S14. FT-IR spectrum of TPE.

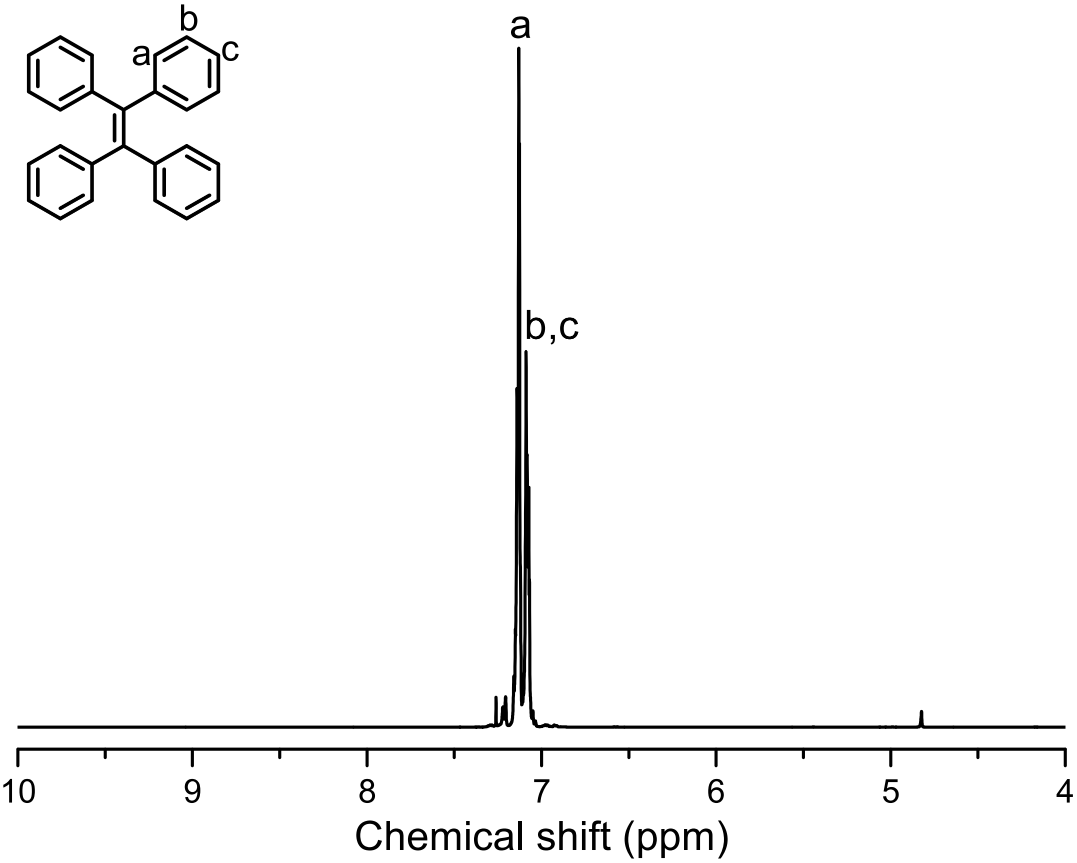

Figure S15. ^{1}H NMR spectrum of TPE.

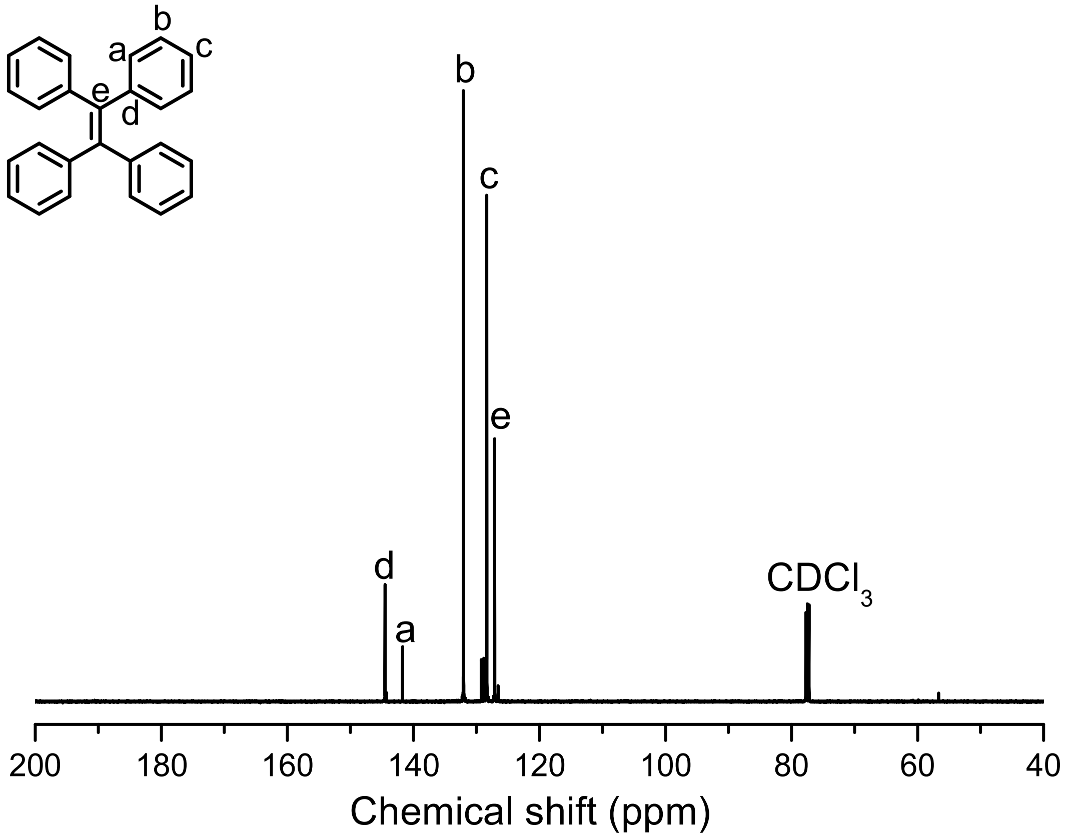

Figure S16. ^{13}C NMR spectrum of TPE.

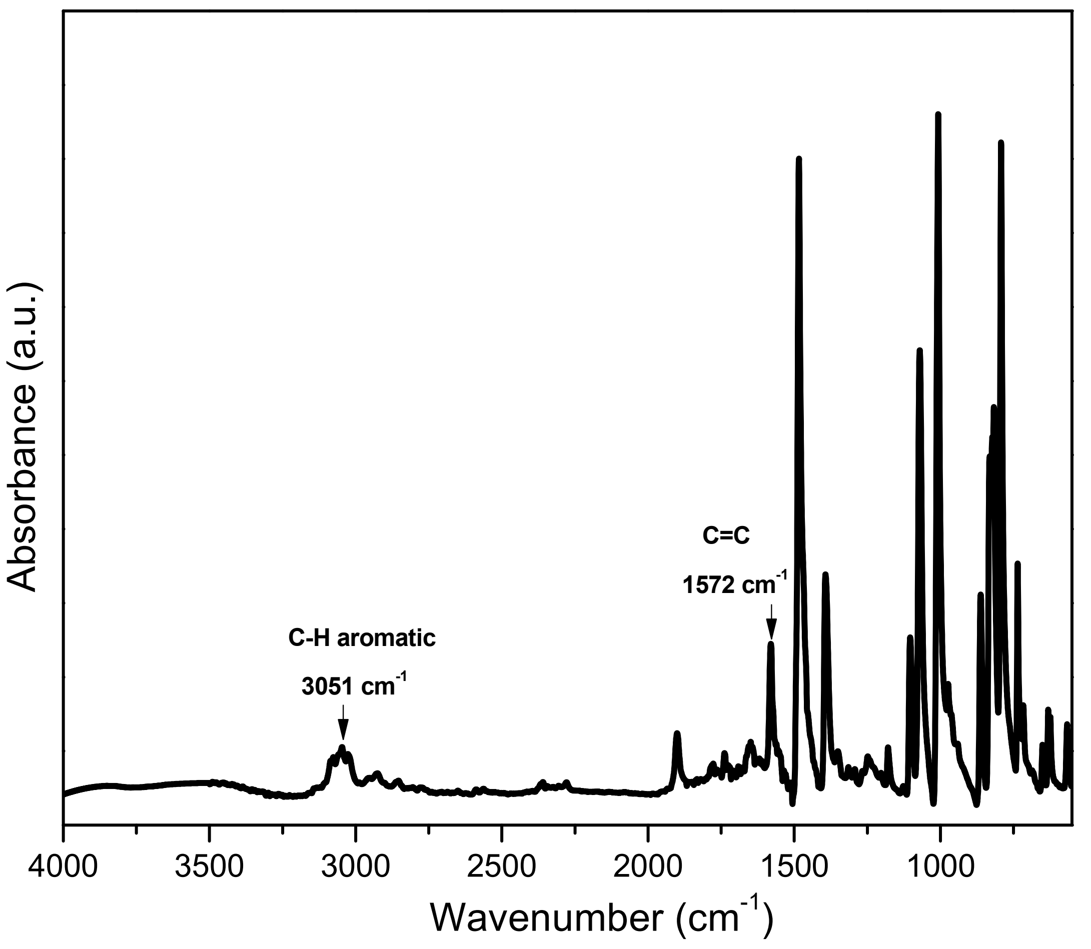

Figure S17. FT-IR spectrum of TPE-Br_4.

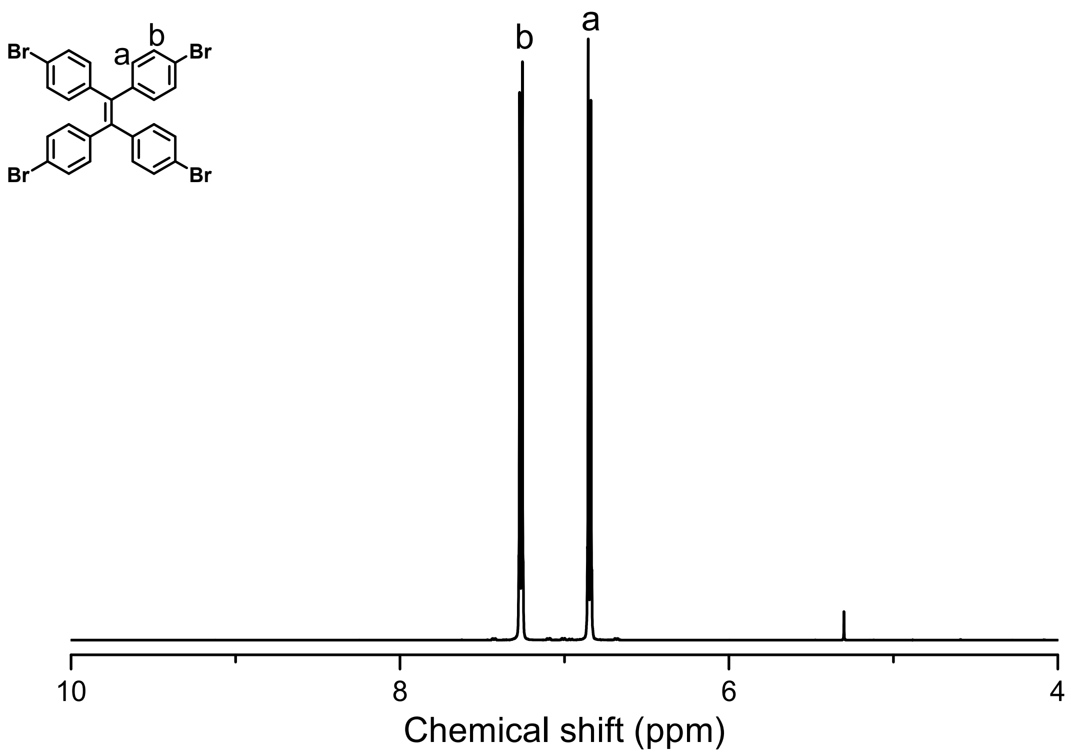

Figure S18. ^{1}H NMR spectrum of TPE-Br_4.

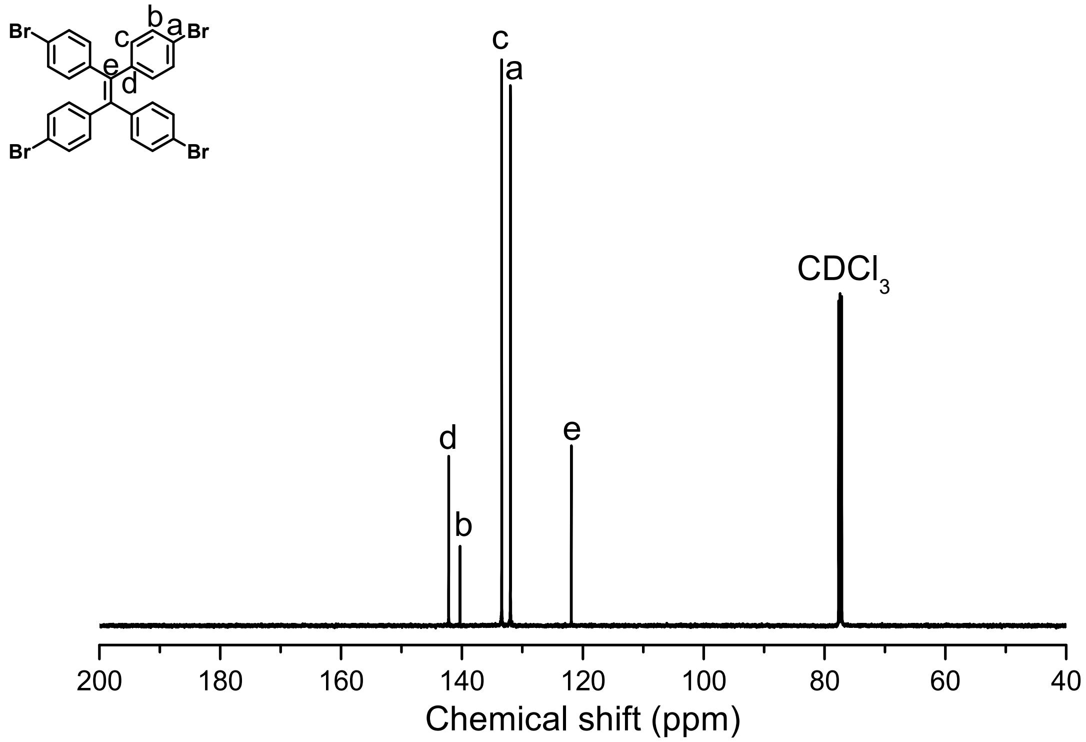

Figure S19. ^{13}C NMR spectrum of TPE-Br_4.

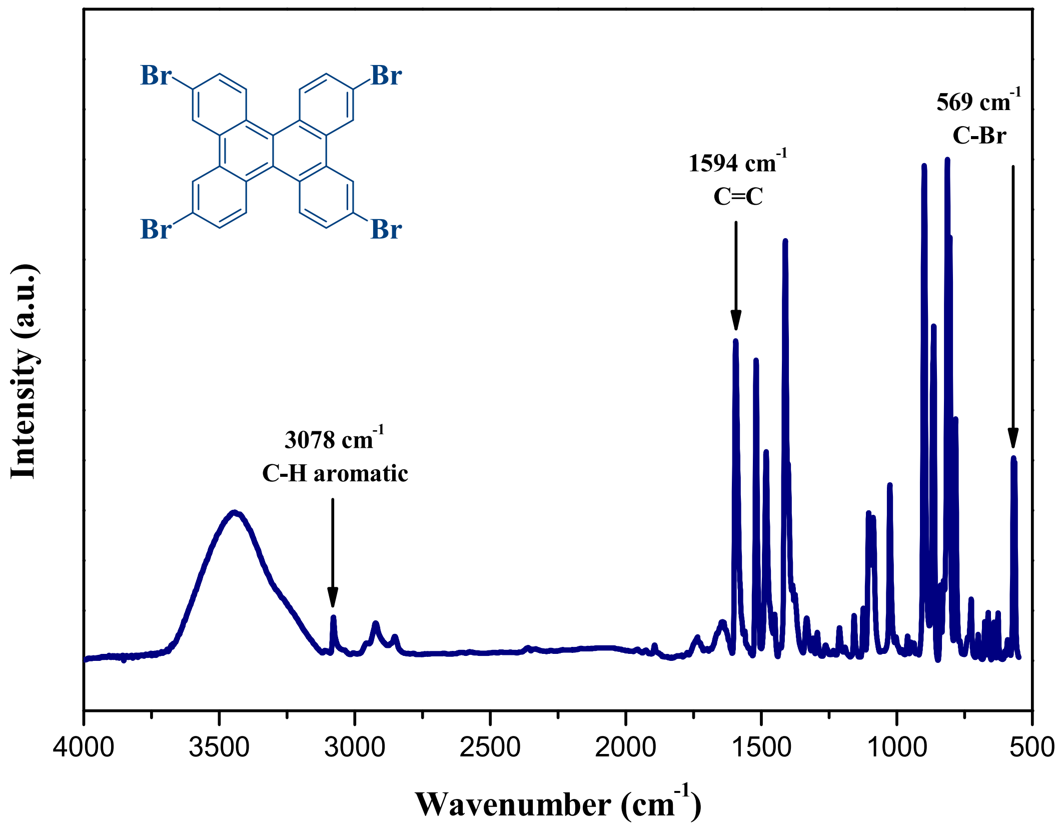

Figure S20. FTIR spectrum of TBN-Br_4.

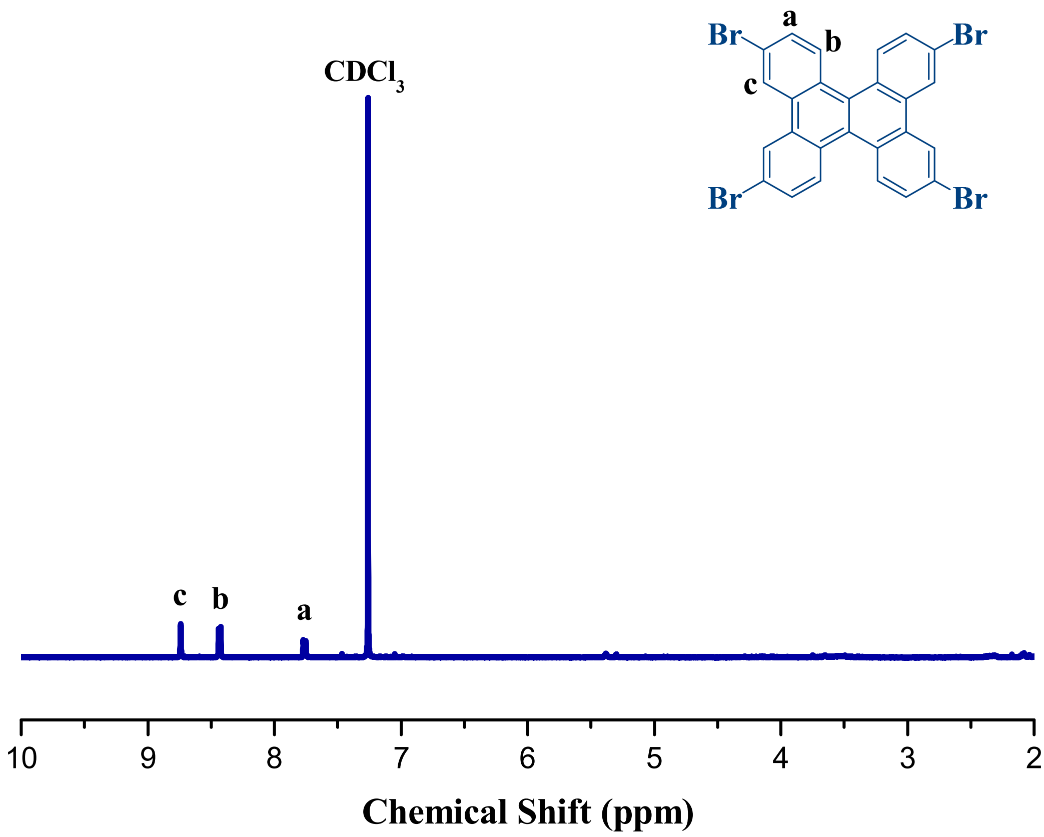

Figure S21. ^{1}H-NMR spectrum of TBN-Br4.

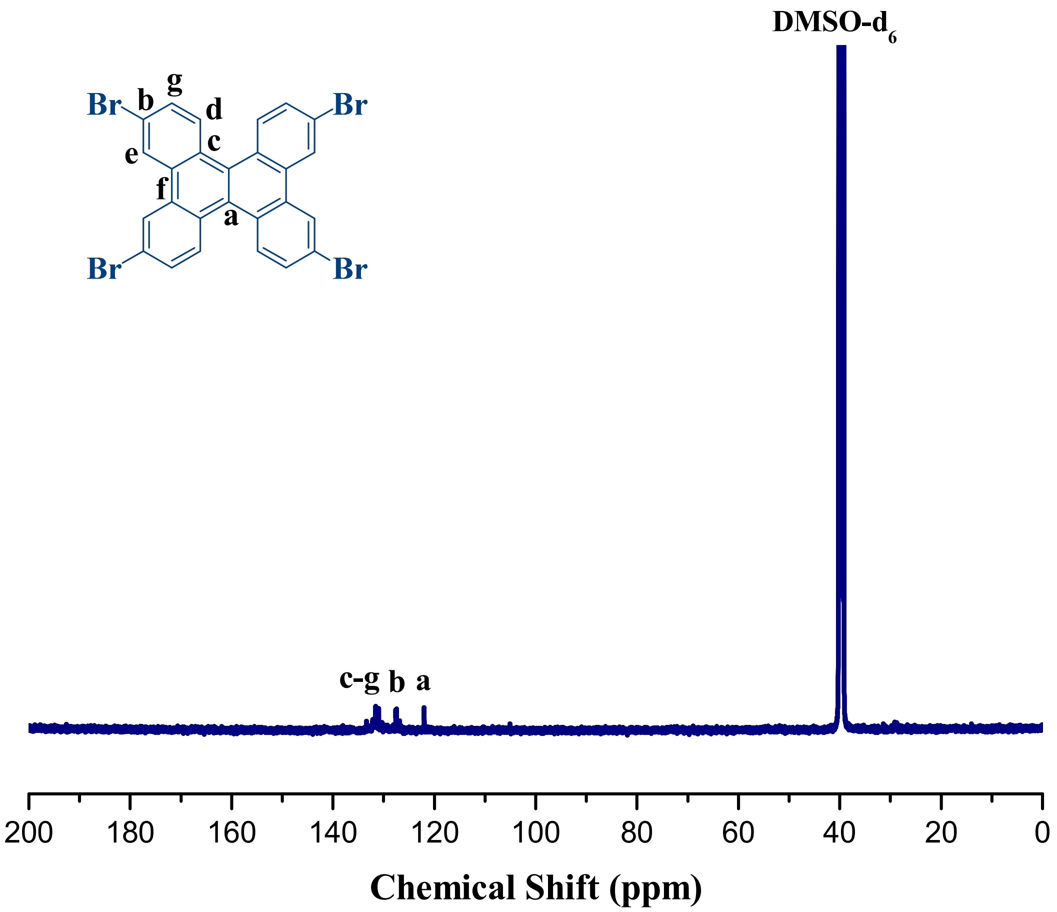

Figure S22. ^{13}C-NMR spectrum of TBN-Br4.

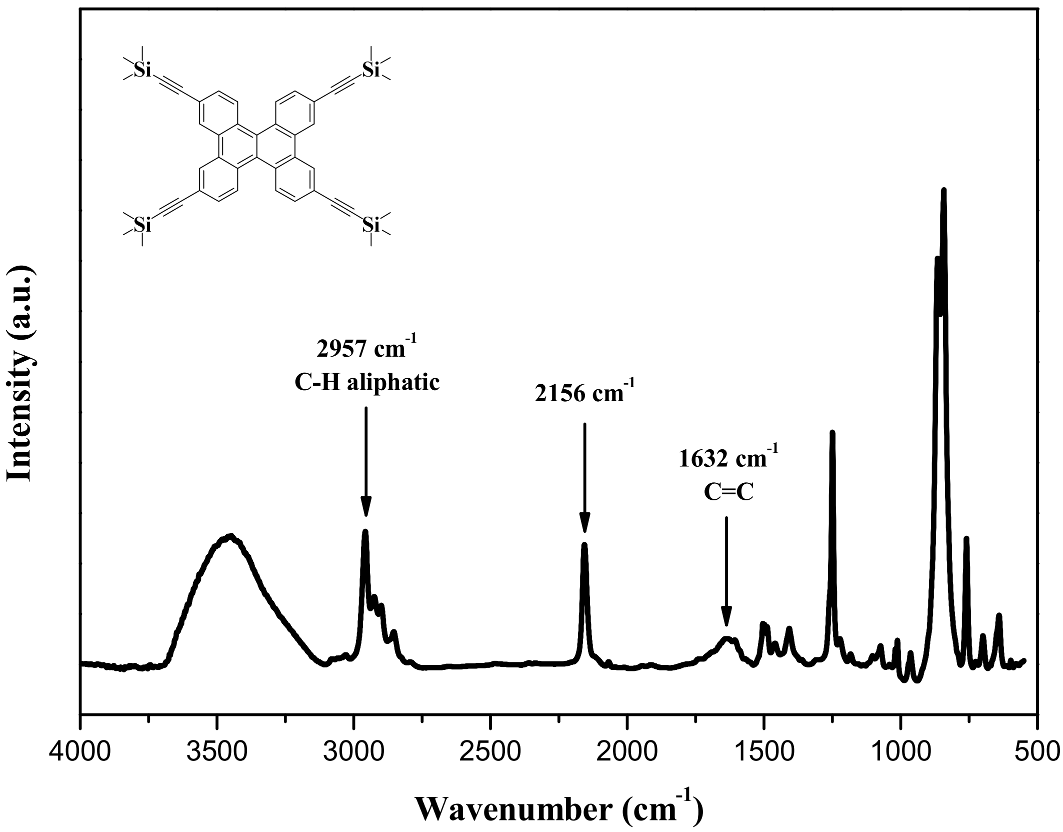

Figure S23. FTIR spectrum of TBN-TMS.

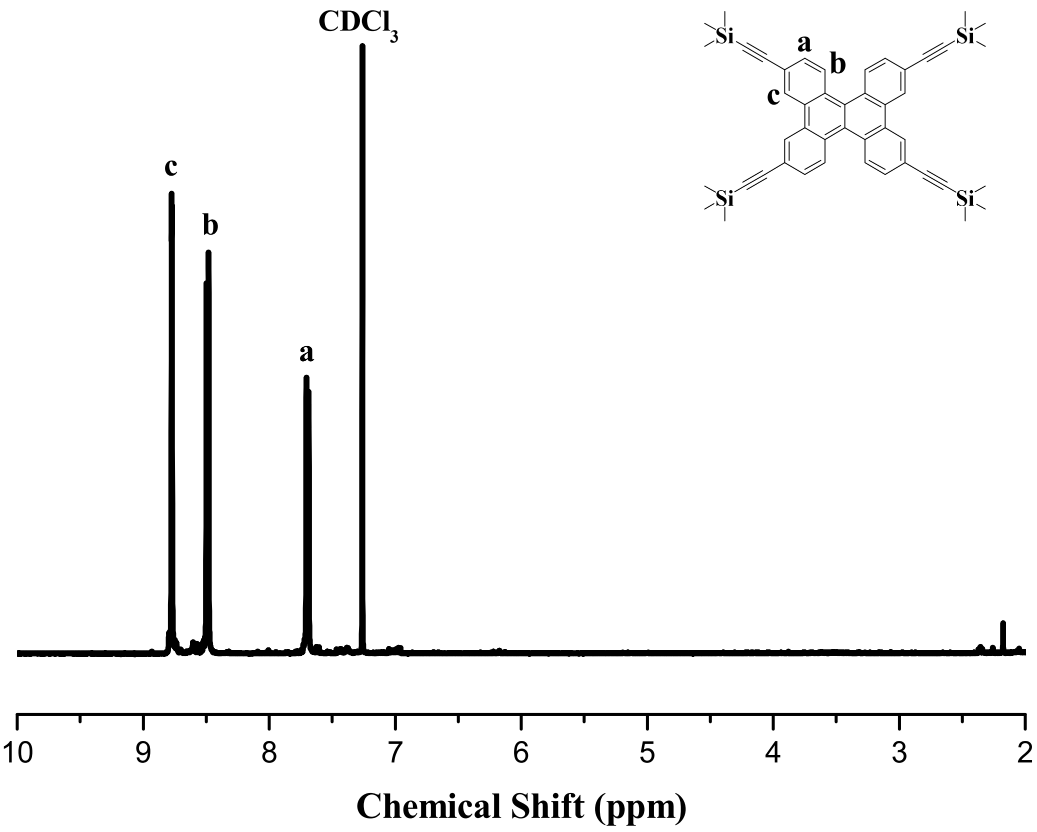

Figure S24. ^{1}H NMR spectrum of TBN-TMS.

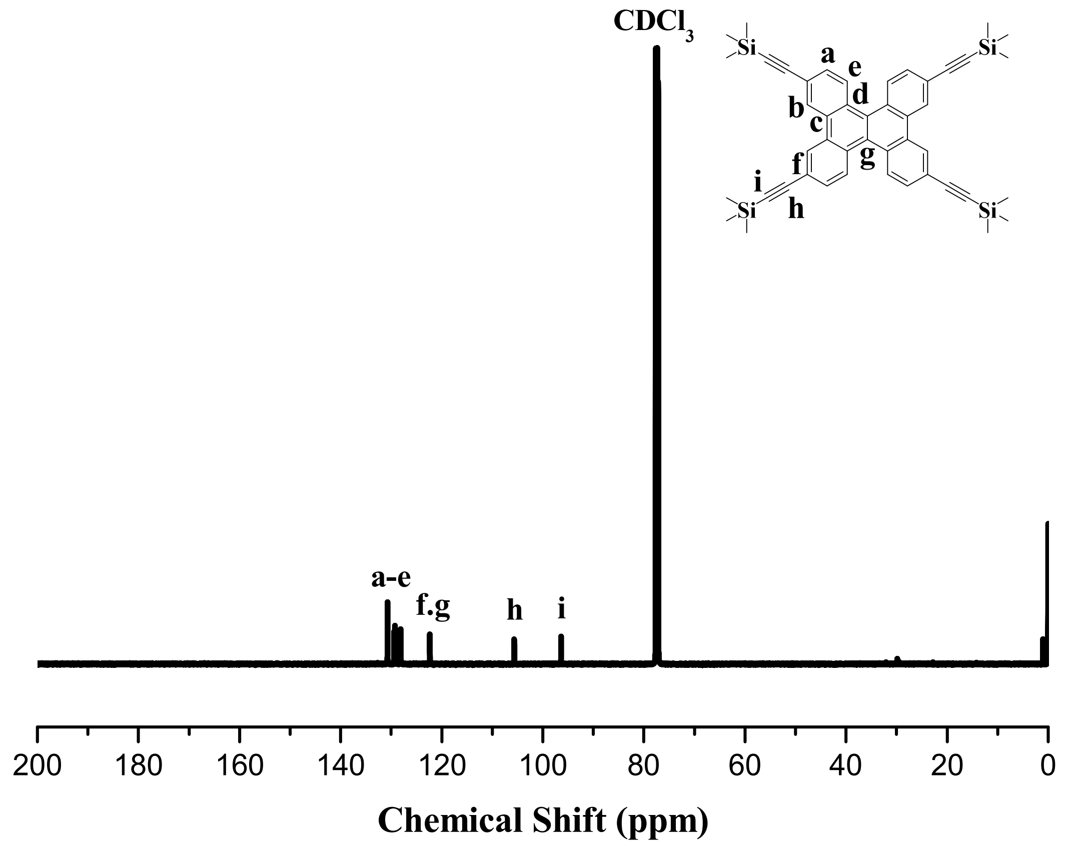

Figure S25. ^{13}C NMR spectrum of TBN-TMS.

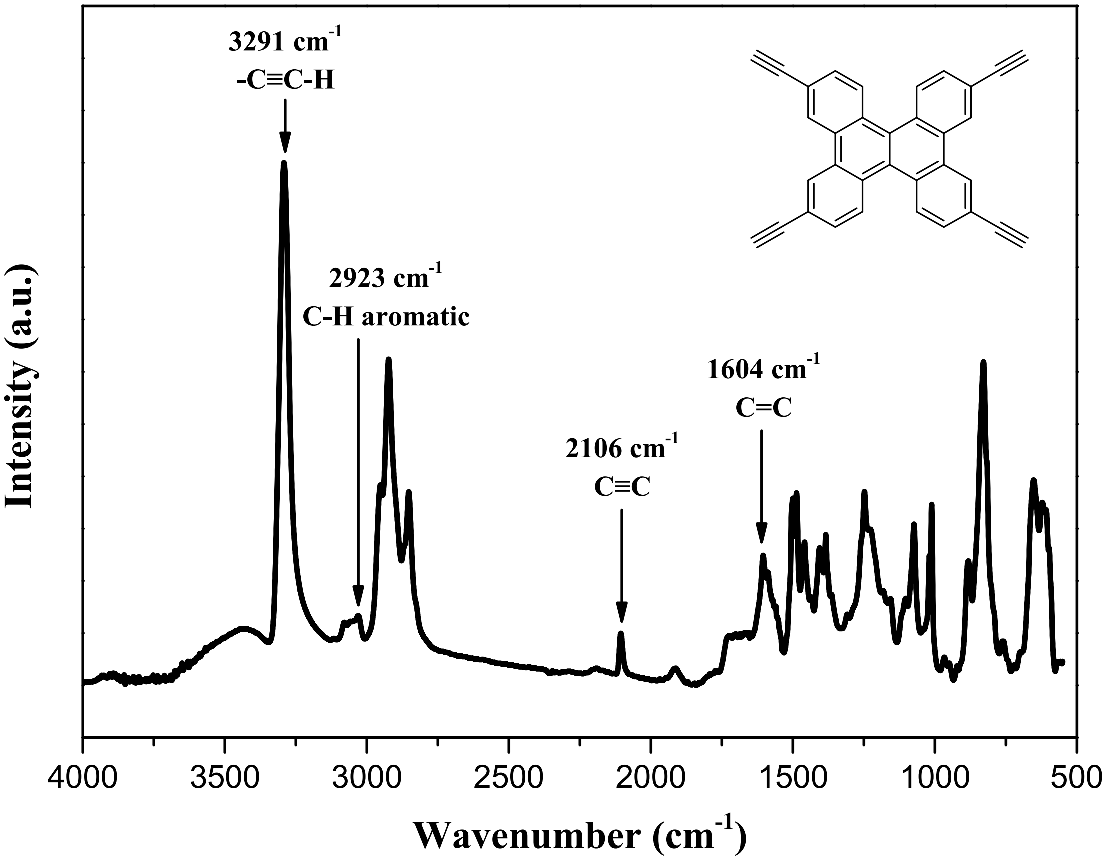

Figure 26. FTIR spectrum of TBN-T.

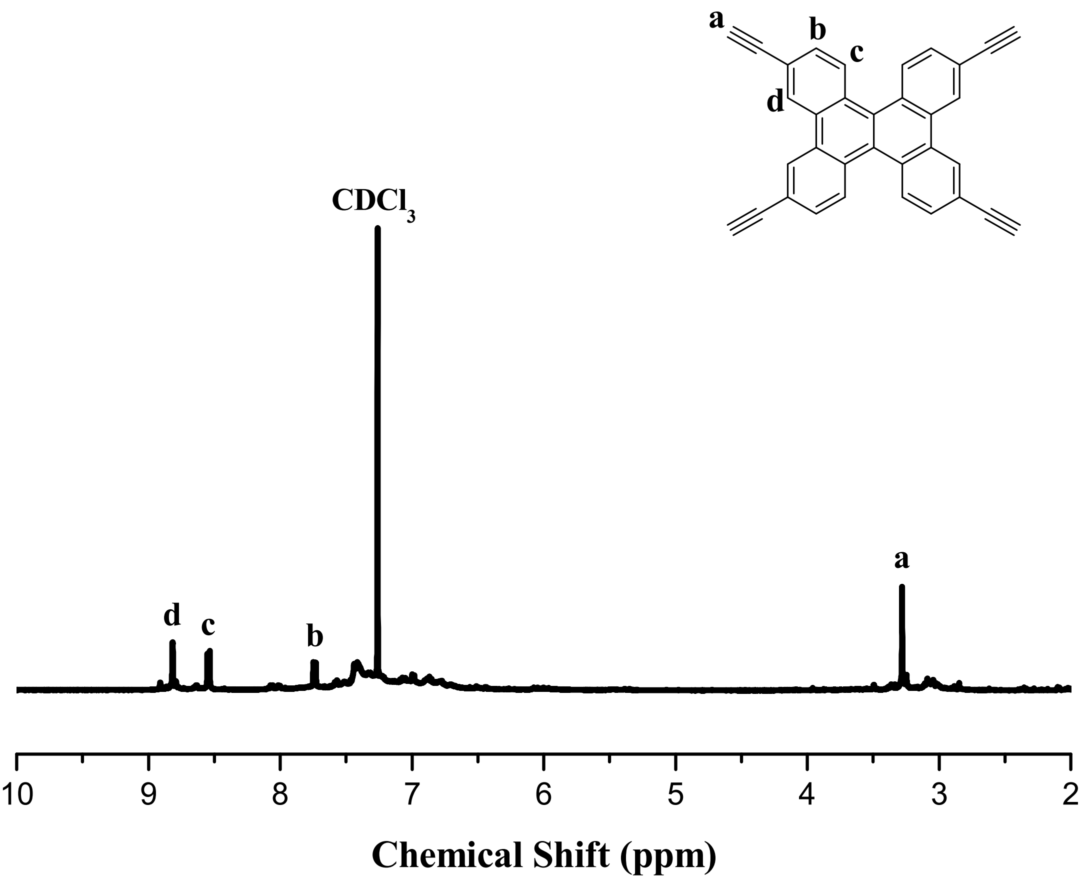

Figure S27. ^{1}H NMR spectrum of TBN-T.

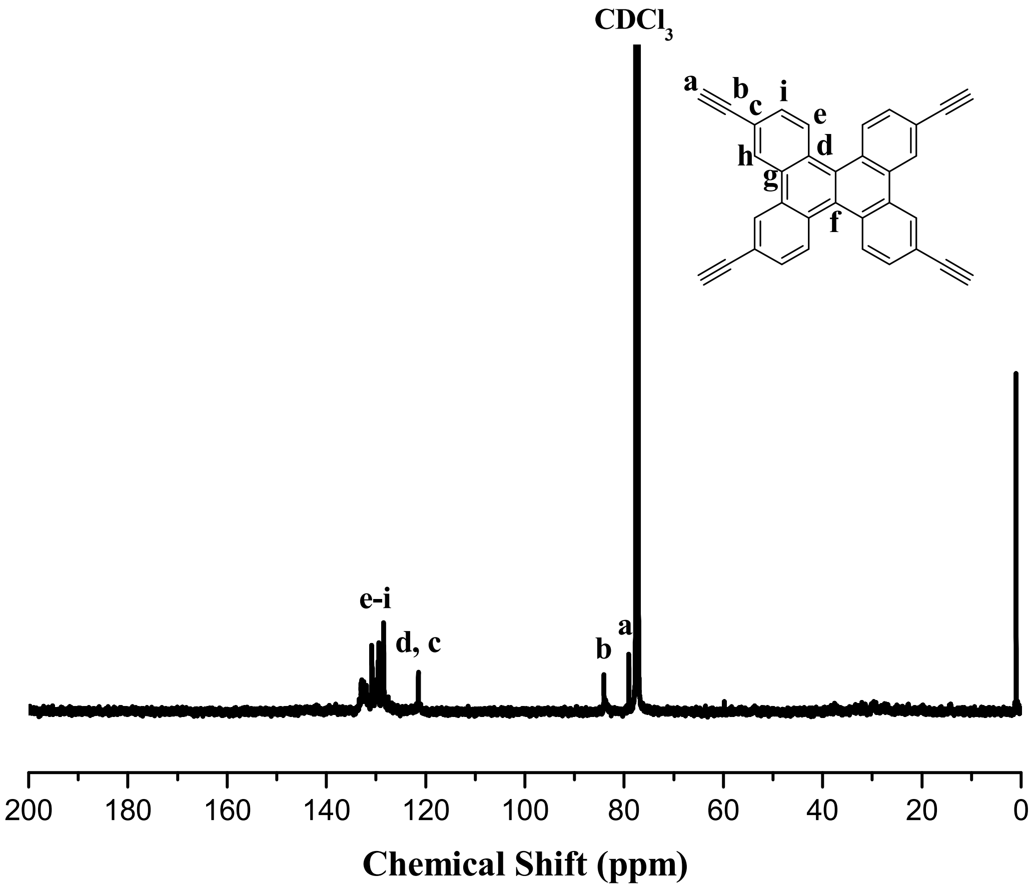

Figure S28. ^{13}C NMR spectrum of TBN-T.

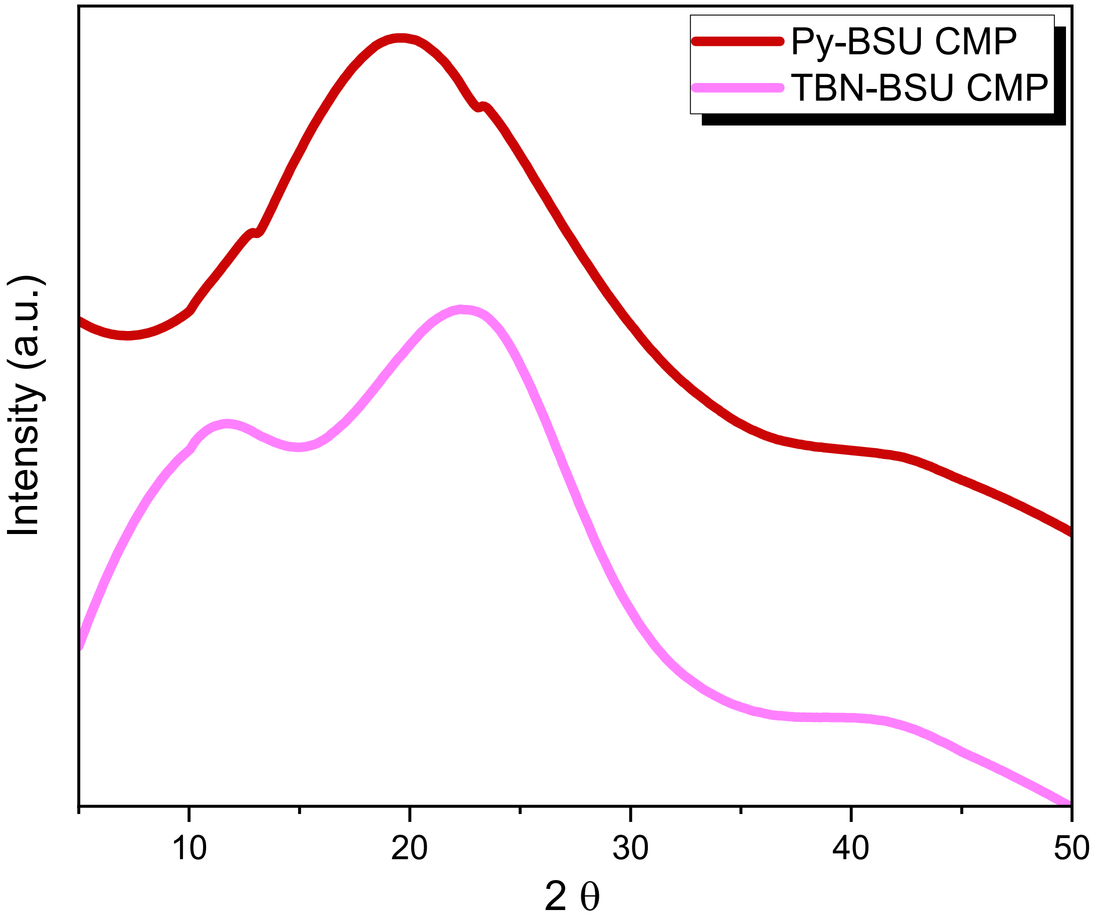

Figure S29. XRD profiles of Py-BSU CMP and TBN-BSU CMP.

(a)

Element	Weight%	Atomic%
C K	78.80	84.67
O K	16.82	13.57
S K	4.38	1.76
Totals	100.00	

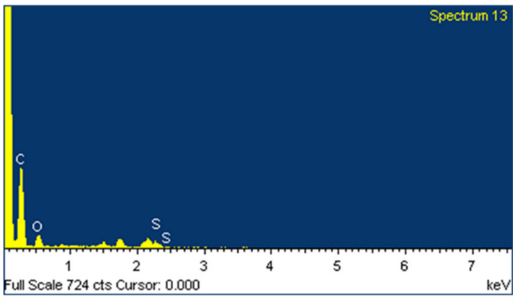

(b)

Element	Weight%	Atomic%
C K	70.90	77.22
O K	26.64	21.78
S K	2.46	1.01
Totals	100.00	

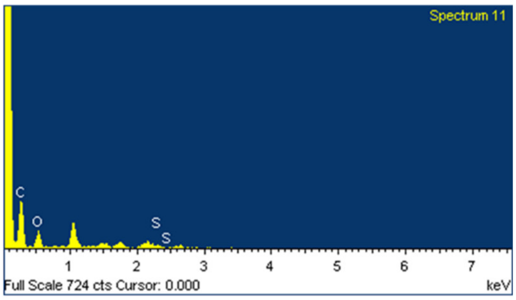

Figure S30. Weight elements contents profiles of (a) Py-BSU CMP and (b) TBN-BSU CMP.

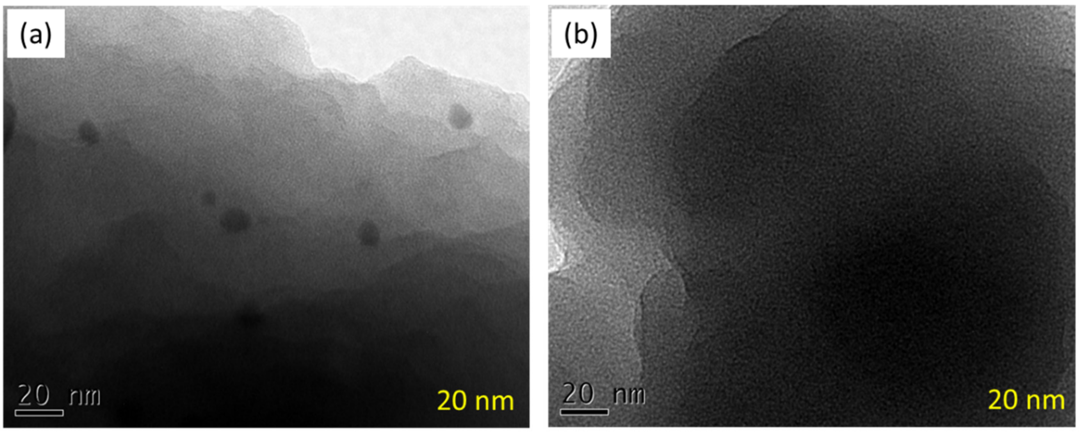

Figure S31. TEM images of Py-BSU CMP and TBN-BSU CMP.

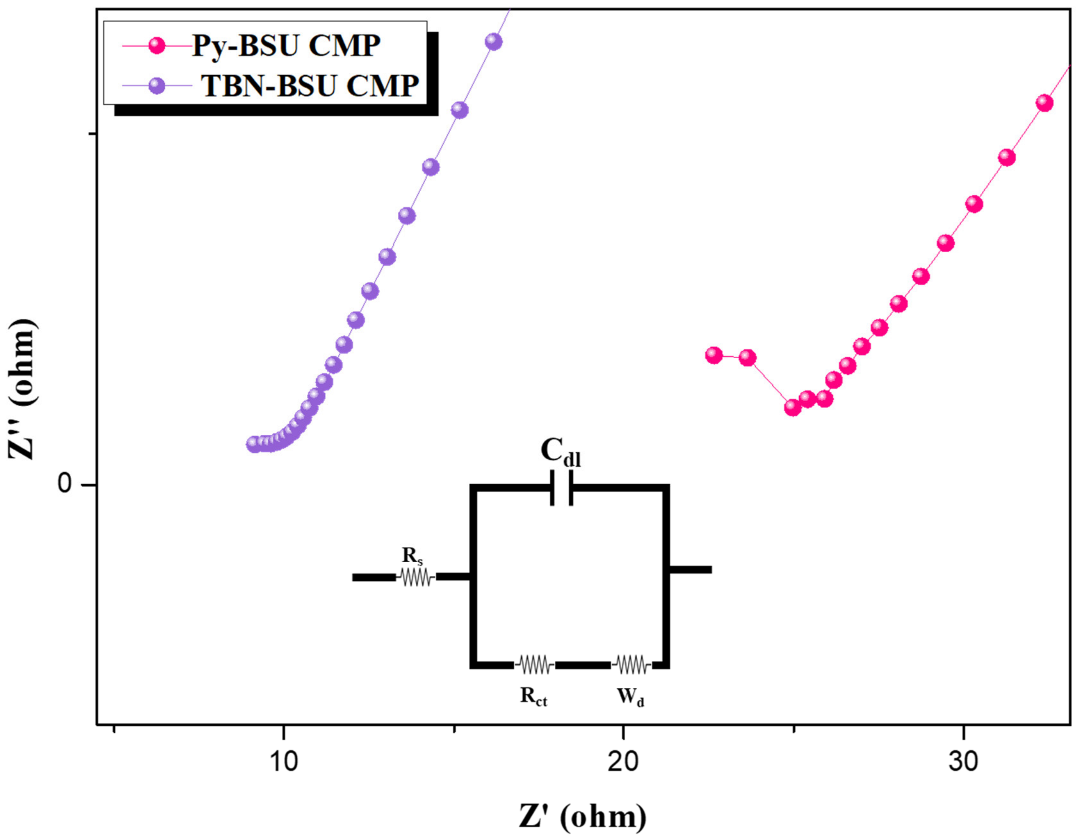

Figure S32. Fitted circuits of the Nyquist plots of Py-BSU CMP and TBN-BSU CMP.
